# Itaconate suppresses neonatal intestinal inflammation via metabolic reprogramming of M1 macrophage

**DOI:** 10.1002/ctm2.70419

**Published:** 2025-07-17

**Authors:** Shuchen Huangfu, Chaoting Lan, Sitao Li, Huijuan Wang, Chun Yan, Yuling Yang, Bowen Tian, Yide Mu, Peizhi Zhao, Yan Tian, Yijia Wang, Wei Zhong, Limei Zhong, Yongyan Shi, Yufeng Liu

**Affiliations:** ^1^ Center for Medical Research on Innovation and Translation Guangzhou First People' s Hospital, the Second Affiliated Hospital of South China University of Technology Guangzhou Guangdong China; ^2^ Department of Pediatric Surgery Guangdong Provincial Key Laboratory of Research in Structural Birth Defect Disease, Guangzhou Women and Children's Medical Center, Guangzhou Medical University, Guangdong Provincial Clinical Research Center for Child Health Guangzhou Guangdong China; ^3^ Department of Pediatrics the Sixth Affiliated Hospital, Sun Yat‐sen University Guangzhou Guangdong China; ^4^ Biomedical Innovation Center the Sixth Affiliated Hospital, Sun Yat‐sen University Guangzhou Guangdong China; ^5^ Department of Anesthesiology Jiangxi Provincial Children's Hospital Nanchang Jiangxi China; ^6^ Department of Laboratory Medicine the Affliated Guangdong Second Provincial General Hospital of Jinan University Guangzhou Guangdong China; ^7^ Division of Neonatology Department of Pediatrics Shengjing Hospital of China Medical University Shenyang Liaoning China

**Keywords:** itaconate, macrophage, metabolic reprogramming, necrotizing enterocolitis, oxidative phosphorylation

## Abstract

**Background:**

Necrotizing enterocolitis (NEC) is a rapidly progressive and severe gastrointestinal disorder in neonates that is marked by an inflammatory cascade initiated by mechanisms that remain incompletely understood, resulting in intestinal necrosis and systemic infections. This study demonstrated that itaconate (ITA) exerts a protective effect in NEC by regulating macrophage reprogramming.

**Methods:**

Changes in ITA expression were investigated using immunofluorescence staining and liquid chromatography‐mass spectrometry, and their effect on immune cell differentiation was verified through single‐cell sequencing. In vivo experiments were performed using *ACOD1* ^−/‐^ and ACOD1^fl/fl^LysM^cre^ NEC mouse models.

**Results:**

We detected changes in ITA expression in clinical NEC samples and confirmed the effect of these changes on immune cell differentiation. In vivo experiments confirmed the therapeutic role of ITA in regulating macrophage differentiation in NEC, and we further investigated the mechanism by which ITA regulates macrophage metabolic reprogramming. The depletion of ITA in NEC correlates with an increased frequency of pro‐inflammatory macrophage polarization, thereby exacerbating intestinal inflammatory injury. Importantly, our in vivo experiments revealed that treatment with 4‐octyl itaconate (4OI) significantly mitigated intestinal symptoms associated with NEC in murine models. Mechanistic investigations showed that 4OI effectively suppressed M1 macrophage polarization by rescuing mitochondrial function and upregulating oxidative phosphorylation in macrophages.

**Conclusions:**

Our results highlight ITA as a metabolic checkpoint of macrophage differentiation in NEC and suggest the therapeutic efficacy of 4OI in NEC.

**Key points:**

- Itaconate alleviates NEC by reprogramming M1 macrophage metabolism
- *ACOD1* deficiency exacerbates NEC severity
- 4OI maintains intestinal barrier integrity.
- 4OI rescues NEC by regulating macrophage mitochondrial activity.

## Graphical abstract

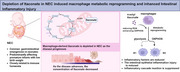

## 1 INTRODUCTION

Necrotizing enterocolitis (NEC) is a prevalent and severe gastrointestinal emergency in neonates that effect premature newborns with low birth weight.^1^ Its defining feature is the rapid emergence of intestinal inflammation, leading to necrosis and systemic infection, which accounts for its high morbidity and mortality rates.^2^ Despite significant advances in neonatal treatment, the pathogenesis of NEC remains unclear and therapeutic options are limited, with surgery being the last resort.^3^ According to the data of a multicentre retrospective study in China, the incidence of NEC with a BELL stage ≥ stage II is 3.3% to 7.6% and increases as the gestational age and birth weight decreases. Despite aggressive medical and surgical interventions, the in‐hospital mortality rate of NEC in children is still 9.4% to 17.6%.^4^ The lack of effective medical interventions highlights the urgent need to elucidate the mechanisms underlying NEC pathophysiology.

Recent data have shown that the immune system plays a central role in the development of NEC, with dysregulated inflammatory responses being critical for the initiation and progression of the disease.^5^ Macrophages are key players in immune response that have great plasticity and are implicated in the inflammatory cascade of NEC.^6^, ^7^ Emerging evidence suggests that metabolic reprogramming of macrophages is a key mechanism that affects their function.^8^, ^9^ This metabolic reprogramming not only affects macrophage energy production and the biosynthesis of metabolic intermediates but also affects their immune regulatory functions.^10^ Depending on their reliance on aerobic glycolysis and oxidative phosphorylation (OXPHOS) for energy production, macrophages can alter between a pro‐inflammatory M1 phenotype, which aggravates tissue damage, and an anti‐inflammatory M2 phenotype, which facilitates tissue repair and resolution of inflammation.^11^ The metabolic flexibility of macrophages represents a potential therapeutic target for controlling inflammation in NEC.

ITA has been widely studied as a metabolite with well‐documented anti‐inflammatory and immunomodulatory properties. It is produced by fumarate decarboxylase 1 (*ACOD1*, also known as immune‐responsive gene 1, *Irg1*), which catalyses the decarboxylation of cis‐aconitine.^12^ ITA is a crucial regulator of immune responses, particularly in macrophages, where it exerts significant anti‐inflammatory effects.^13^, ^14^ Its mechanism of action is multifaceted. It can competitively inhibit succinate dehydrogenase (SDH), thus preventing the release of interleukin‐1β (IL‐1β) and reactive oxygen species (ROS) caused by the accumulation of succinate. It can also activate the Nrf2 signalling pathway, thereby inhibiting NF‐κB signalling transduction. Moreover, ITA can enhance the antioxidant capacity of cells by modifying the KEAP1 protein.^15^, ^16^, ^17^ Furthermore, ITA significantly influences other immune cells. Tomlinson et al. showed that ITA from neutrophils protects the lung from excessive inflammation by inhibiting glycolytic and oxidative bursts of neutrophils during *Staphylococcus*. *aureus* infection.^18^ A previous study has shown that ITA can regulate the metabolic reprogramming of macrophages by inhibiting SDH and other means, influencing their polarization and function, thereby playing a significant role in many inflammatory diseases.^19^ The interrelated mechanism between metabolism and immunity makes ITA an promising target for the treatment of inflammatory disorders.

The function of ITA in regulating inflammation has been studied in various diseases. Although recent studies have shown that ITA protects the integrity of the intestinal epithelial barrier by enhancing autophagic flow and lysosomal function of intestinal epithelioid cells in NEC,^20^ its role in the intestinal immune microenvironment of NEC remains unclear. Recent studies have regarded metabolic reprogramming of targeted immune cells as a therapeutic strategy for alleviating inflammatory damage.^21^ Given the central role of macrophages in NEC and the established anti‐inflammatory properties of ITA, we postulated that ITA confers protection against NEC by modulating macrophage polarization and metabolic activity. In addition, oxidative stress and redox imbalances are known to contribute to the pathogenesis of NEC; however, extent to which ITA regulates redox homeostasis and mitochondrial function in macrophages during NEC is unclear. Understanding the role of ITA in metabolism and function of macrophages will help advance our knowledge of NEC pathogenesis and lay the groundwork for novel therapeutic approaches.^22^

This study aimed to investigate whether ITA induces changes in the metabolic phenotype of macrophages by regulating their mitochondrial function, thereby exerting therapeutic effects against NEC.

## 2 RESULTS

### 2.1 ITA deficiency occurs in the development of NEC

**FIGURE 1:**
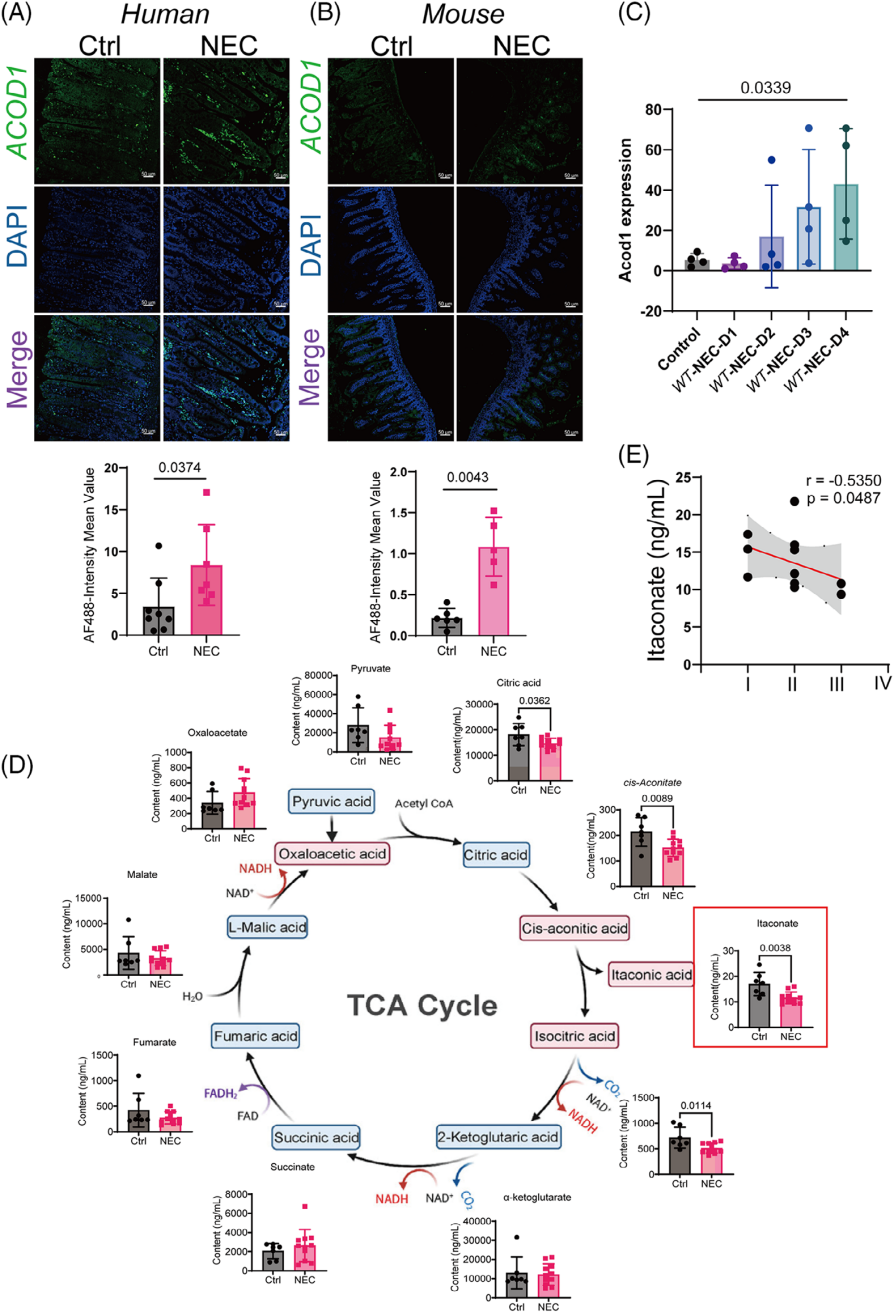
The absence of itaconate (ITA) is present in the development of necrotizing enterocolitis (NEC). (A‐B) Representative confocal images and quantification of fluorescent intensity of ACOD1 immuno‐stained ileal sections of human (*n* = 8 per group) (A), mouse (*n* = 5 per group) (B). (C) Expression levels of ACOD1 in mice subjected to varying durations of modelling (*n* = 4). (D) Relative abundance of TCA cycle metabolites (ITA, isocitrate, α‐ketoglutarate, succinate, malate, pyruvate, citrate, and cis‐aconitate) was measured by metabolomics in control (*n* = 7) and NEC (*n* = 11) group. a.u., arbitrary units based on MS peak area, Data are presented as mean ± SEM. Unpaired t test, two‐tailed. (E) Analysis of the correlation between NEC clinical disease staging (BELL Score) and ITA Content, Data are presented as mean ± SEM. Unpaired *t*‐test, two‐tailed (D). Wilcoxon signed‐rank test, Two tailed (A B C). Pearson's correlation (E). *p*‐value as shown in Figure.

To investigate the changes in itaconate biosynthesis during NEC, we analysed *ACOD1* expression in the intestines of humans and animals with and without NEC. This study included patients who were clinically and pathologically diagnosed with NEC according to the BELL staging system. *ACOD1* expression levels were elevated in the intestinal tissues of patients with NEC and mice (Figure 1A,B) progressively increasing from day one to four after NEC induction (Figure 1C), which indicates its upregulation in response to inflammation. Metabolite analysis revealed a significant decrease in ITA levels in the peripheral blood of patients with NEC relative to healthy controls with citrate, cis‐aconitate, and isocitrate levels also being affected (Figure 1D). A strong negative association was observed between ITA levels and NEC severity as classified the Bell classification (Figure 1E). Although the expression of genes that regulate ITA synthesis increased, the level of ITA and the substrates for its synthesis decreased with disease progression, suggesting that ITA plays a role in attenuating NEC‐associated inflammation.

### 2.2 *ACOD1* deficiency aggravates inflammatory injury in NEC

**FIGURE 2:**
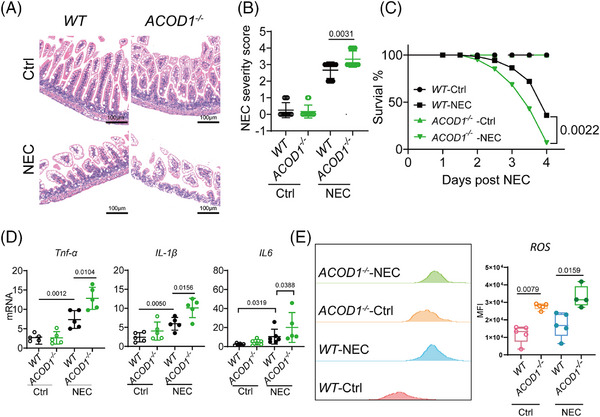
*ACOD1* deficiency aggravates inflammatory injury in necrotizing enterocolitis (NEC). (A) Representative images of H&E‐stained of the intestinal epithelial tissue in *WT* mice and *ACOD1^−/−^* mice. (B) Severity score and of *WT* mice and *ACOD1^−/−^* mice in NEC, (*n* = 12 per group). (C) survival curve of *WT* mice and *ACOD1^−/−^* mice in NEC, (*n* = 20 per group). (D) Quantitative measurement of mRNA levels of inflammatory factors IL‐6, IL‐1β, and TNF‐α, (*n* = 5 per group). (E) Flow cytometry analysis of ROS levels (left) and quantification of mean fluorescence intensity (MFI) (right) in *WT* and *ACOD1* ^−/−^ mice under control and NEC conditions, (*n* = 5 per group). Data represent mean ± SD, Wilcoxon signed‐rank test, Two‐tailed (B D E). Log‐rank test (C). *p*‐value as shown in Figure.

We examined the protective function of ITA in NEC using *ACOD1* knockout (*ACOD1* ^−/−^) mice randomly assigned to a control or NEC model group. Haematoxylin and eosin (HE) staining of intestinal epithelial tissue from *ACOD1^−/‐^* mice revealed significantly greater epithelial damage during NEC progression (Figure 2A). Histological analysis indicated markedly increased tissue damage scores in necrotic lesions of *ACOD1^−/−^* mice compared with controls (Figure 2B). Survival analysis further demonstrated a significantly lower survival rate in *ACOD1^−/−^* mice compared with wild‐type mice following NEC induction, consistent with the increased disease severity scores (Figure 2C). Additionally, *ACOD1^−/−^* mice exhibited heightened inflammatory responses compared with wide‐type mice, having significantly elevated levels of the pro‐inflammatory cytokines *IL‐6*, *IL‐1β*, and *TNF‐α* (Figure 2D). Flow cytometry analysis revealed significantly elevated ROS levels in *ACOD1^−/−^* mice under both physiological and experimental conditions (Figure 2E). Our findings suggest that the lack of ITA leads to higher ROS levels and exacerbates NEC‐induced inflammation.

### 2.3 *ACOD1* deficiency promotes the proinflammatory polarization of macrophages during NEC

**FIGURE 3:**
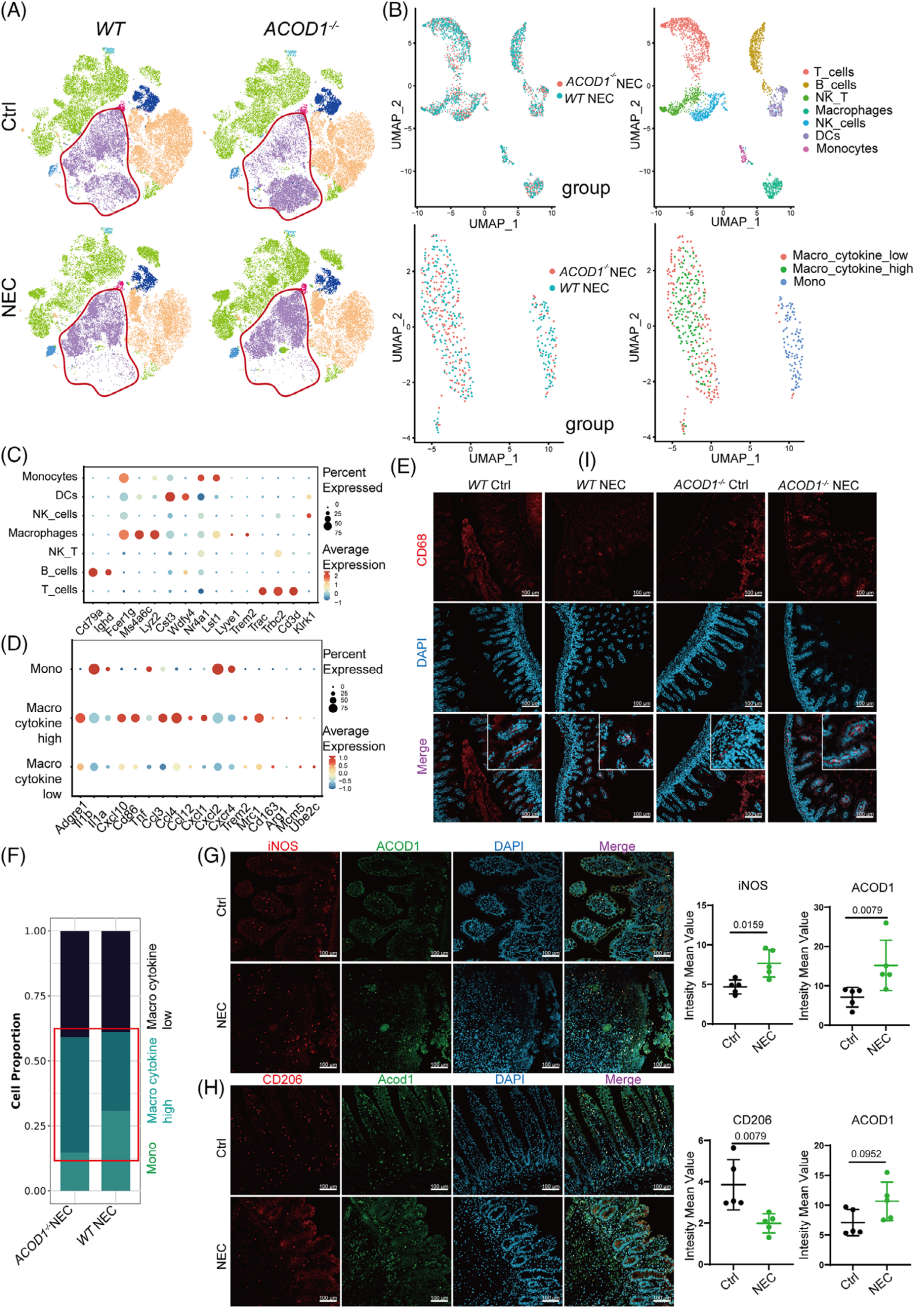
*ACOD1* deficiency promotes the proinflammatory polarization of macrophages during necrotizing enterocolitis (NEC). (A) UMAP plot of dimensionality reduction analysis of flow cytometry data for immune cells isolated from intestinal tissue of the *WT* and *ACOD1* ^−/−^ mice in the negative control group and NEC group, *WT* Ctrl (*n* = 5), *WT* NEC (*n* = 6), *ACOD1* ^−/‐^ Ctrl (*n* = 4), *ACOD1* ^−/−^ NEC (*n* = 3). Clusters are coloured as in Figure S1C. (B) UMAP plots of immune cells from NEC *WT* and *ACOD1* ^−/−^ mice, data from scRNA‐sequencing. (C) The expression of specific marker genes in each kind of immune cells populations was determined. The size of the dots indicates the percentage of cells expressing the gene of interest, while the intensity of the colour indicates expression levels. (D) The expression of M1‐like and M2‐like marker genes in each macrophage subpopulation. The size of the dots and intensity of colours as in B. (E) Representative confocal images of fluorescent intensity of CD68 in intestinal tissues of *WT* and *ACOD1* ^−/−^ mice. (F) The proportion of macrophage subpopulations from NEC *WT* and *ACOD1* ^−/−^ mice. (G) Representative confocal images of fluorescent intensity and quantitative statistical analysis of iNOS and ACOD1 in intestinal tissues of NEC patients and healthy control (*n* = 5 per group). (H) Immunofluorescence analysis of CD206 and ACOD1 in intestinal tissues of NEC patients and healthy control (*n *= 5 per group). Data represent mean ± SD, Wilcoxon signed‐rank test, Two‐tailed (G H). *p*‐value as shown in Figure.

We evaluated the effect of *ACOD1* deficiency on different immune cell populations during NEC using intestinal tissues from both *WT* and *ACOD1^−/−^* mice with and without NEC. The UMAP dimensionality reduction analysis was performed using multiparameter flow cytometry data from intestinal immune cells. The gating strategy used in the analysis is shown in Figure S1A. UMAP dimensionality reduction analysis was performed based on the results of multicolour flow cytometry analysis of specific immune‐cell population marker features (Figure S1B). The results revealed that the presence or absence of *ACOD1* significantly influenced macrophage infiltration in both control and NEC groups (Figure 3A). Statistical analysis revealed that *ACOD1* loss significantly enhanced macrophage infiltration in intestinal tissues compared with that in the *WT*‐NEC group (Figure S1C). The absence of *ACOD1* in the intestinal tissue led to a marked reduction in the population of myeloid‐derived suppressor cells (MDSCs) (Figure S1B), which maintain intestinal immune tolerance. These results indicate that *ACOD1* deficiency induces an amplified inflammatory response in NEC mice.

We investigated the effect of ITA on immune cell infiltration in NEC mice by performing single‐cell omics analysis of intestinal tissues from *WT* and *ACOD1^−/‐^* NEC mice. We identified multiple immune cell categories (Figure S2A,B). We performed single‐cell sequencing to identify markers following labelling with a specific probe. Genes exhibiting elevated expression levels in macrophages included *Fcer1g*, *Ms4a6c*, and *Lyz2* (Figure 3B,C, Figure S2C). Two macrophage subpopulations distinguished by their cytokine expression levels were identified: one with high expression, typically associated with M1 macrophage activation, and the other with low expression linked to immunosuppressive M2 macrophages (Figure 3B,D). Immunofluorescence analysis showed increased expression of CD68, a macrophage marker, in the intestinal epithelium of *ACOD1^−/−^* NEC mice compared with *WT* mice (Figure 3E, Figure S2E). According to the results of single‐cell analysis, the proportion of macrophages exhibiting elevated cytokine expression was markedly higher in *ACOD1^−/−^* NEC mice than in *WT* mice (Figure 3F). Flow cytometry further confirmed a significant increase in the number of pro‐inflammatory M1 macrophages in *ACOD1^−/−^* NEC mice (Figure S2D). Immunofluorescence analysis of small intestine revealed an increase in iNOS and Acod1 (Figure 3G) and decrease in the CD206 level in the NEC group (Figure 3H). The results of the in vivo macrophage differentiation induction experiments showed that 4‐octyl itaconate (4OI) inhibited the M1 phenotype differentiation of THP‐1 cells (Figure S2F).

### 2.4 *ACOD1* in macrophages protects mice from NEC

**FIGURE 4:**
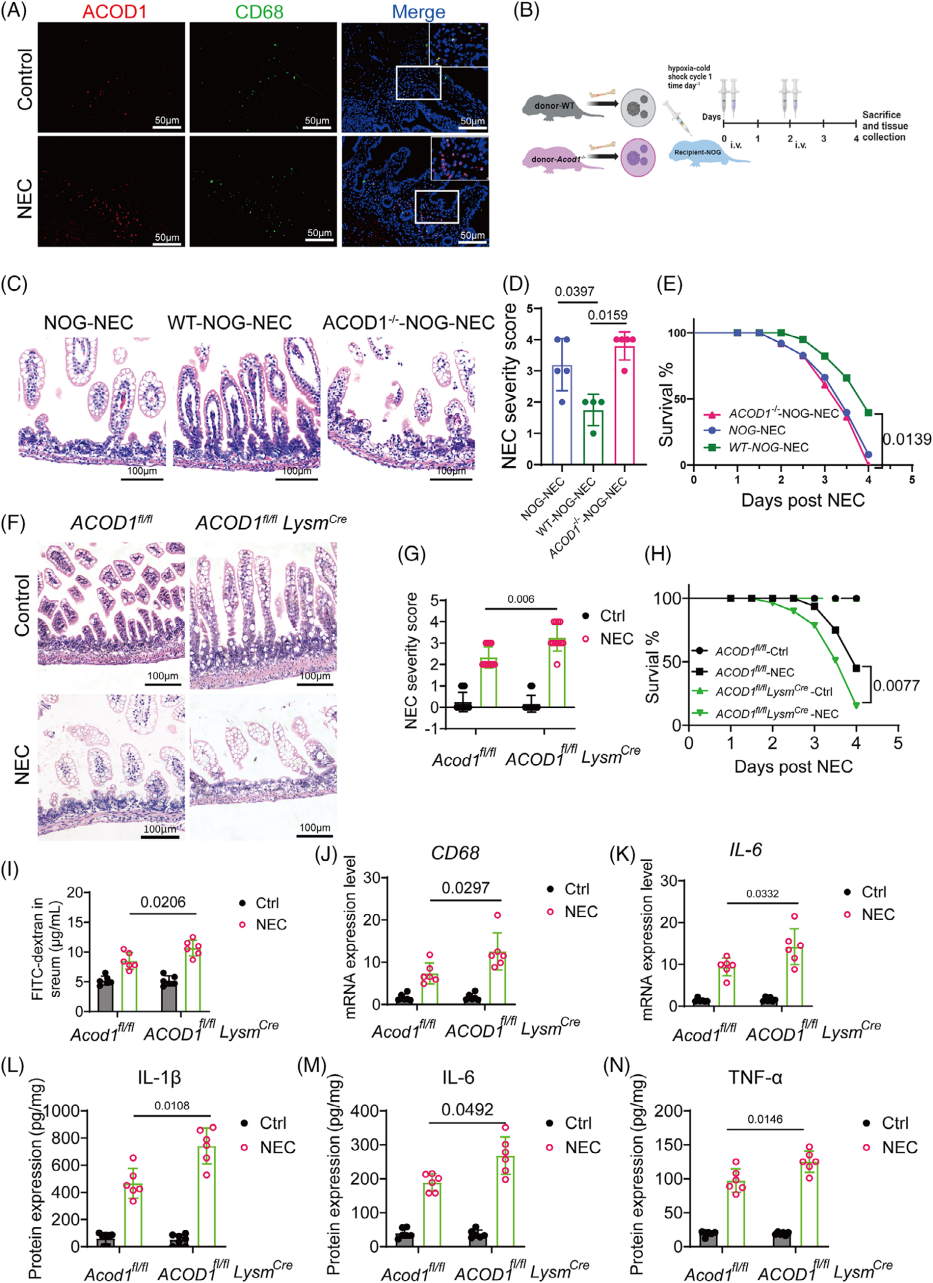
*ACOD1* in macrophages protects mice from necrotizing enterocolitis (NEC). Representative confocal images of co‐localized in human intestinal tissue samples from NEC patients and controls. Red fluorescence represents ACOD1, while green fluorescence represents CD68. (B) Schematic representation of the experimental model for macrophage transplantation. (C) Representative images of H&E‐stained of the intestinal epithelial tissue in NEC NOG mice which received *WT* mice macrophage or *ACOD1* mice macrophage (D) Severity score, NOG‐NEC (*n* = 5), *WT*‐NOG‐NEC (*n* = 4), *ACOD1^−/−^‐*NOG‐NEC (*n* = 5). (E) Survival curve, the experimental groups were organized as in D. (F) Representative images of H&E‐stained of the intestinal epithelial tissue in *ACOD1^fl/fl^* and *ACOD1^fl/fl^ LysM^cre^* mice random allocation in the NEC group and control group. (G) Severity score and survival curve (H) of *ACOD1^fl/fl^* and *ACOD1^fl/fl^ LysM^cre^* mice, *ACOD1^fl/fl^* Ctrl, *ACOD1^fl/fl^* NEC, *ACOD1^fl/fl^ LysM^cre^* Ctrl, *ACOD1^fl/fl^ LysM^cre^* NEC (*n* = 12 per group). (I) Quantification of FITC‐labelled dextran levels in the plasma of mice across different experimental groups (n = 6 per group). (J) mRNA expression of *CD68* and *IL‐6*. (K) in the intestinal epithelial tissue of mice across different experimental groups, (*n* = 12 per group). (L) Protein expression of IL‐6, IL‐1β (M), and TNF‐α (N) in the intestinal epithelial tissue of mice across different experimental groups (*n* = 12 per group). Data represent mean ± SD, Wilcoxon signed‐rank test, Two‐tailed (D); Unpaired *t‐*test, Two‐tailed (G I‐N); Log‐rank test (E and H). *p*‐value as shown in Figure.

Immunofluorescence results further showed the co‐localization of *ACOD1* and *CD68*, suggesting a close association between *ACOD1* expression and macrophages. (Figure 4A). Therefore, we further investigated the role of macrophage‐derived ITA in limiting NEC‐induced inflammatory damage. We transplanted macrophages isolated from *WT* and *ACOD1^−/−^* mice into *NOD/SCID/IL2Rγnull* mice (*NOG*), followed by NEC induction (Figure 4B). H&E staining revealed that *NOG* mice that received *ACOD1^−/−^* macrophages exhibited intestinal epithelial damage comparable to that in untreated *NOG* mice (Figure 4C). In contrast, *NOG* mice that received *WT* macrophages exhibited a marked decrease in epithelial damage and lower mortality rates (Figure 4D,E).

We established conditional *ACOD1*‐knockout mice (*ACOD1^fl/fl^LysM^cre^*) using myeloid‐specific lysosomal‐M (*LysM*)‐Cre mice to investigate the role of *ACOD1* in macrophages during NEC development. NEC were induced in *ACOD1^fl/fl^LysM^cre^* mice and their *ACOD11^fl/fl^* (control) littermates. The results showed that *ACOD11^fl/fl^LysM^cre^* mice had more severe intestinal damage, higher pathological scores (Figure 4F,G), and a significantly lower survival rate during NEC than *ACOD1^fl/fl^* mice (Figure 4H, *p *= 0.0077). FITC‐dextran tests revealed increased intestinal permeability in *ACOD1^fl/fl^ LysM^cre^* NEC mice, indicating that insufficient ITA synthesis by macrophages exacerbated epithelial barrier damage in NEC (Figure 4I). The *ACOD1^fl/fl^LysM^cre^*‐NEC group exhibited significantly elevated expression levels of *CD68* and *IL‐6* increased macrophage migration and infiltration (Figure 4J,K). The protein expression of inflammation‐related genes was considerably elevated in the *ACOD1^fl/fl^LysM^cre^*‐NEC group compared with that in the controls (Figure 4L–N). Collectively, our finding indicated that macrophage‐derived *ACOD1* inhibits excessive inflammatory responses macrophage chemotaxis and NEC infiltration.

### 2.5 *ACOD1* deficiency accelerates the switch of macrophages towards glycolysis in NEC

**FIGURE 5:**
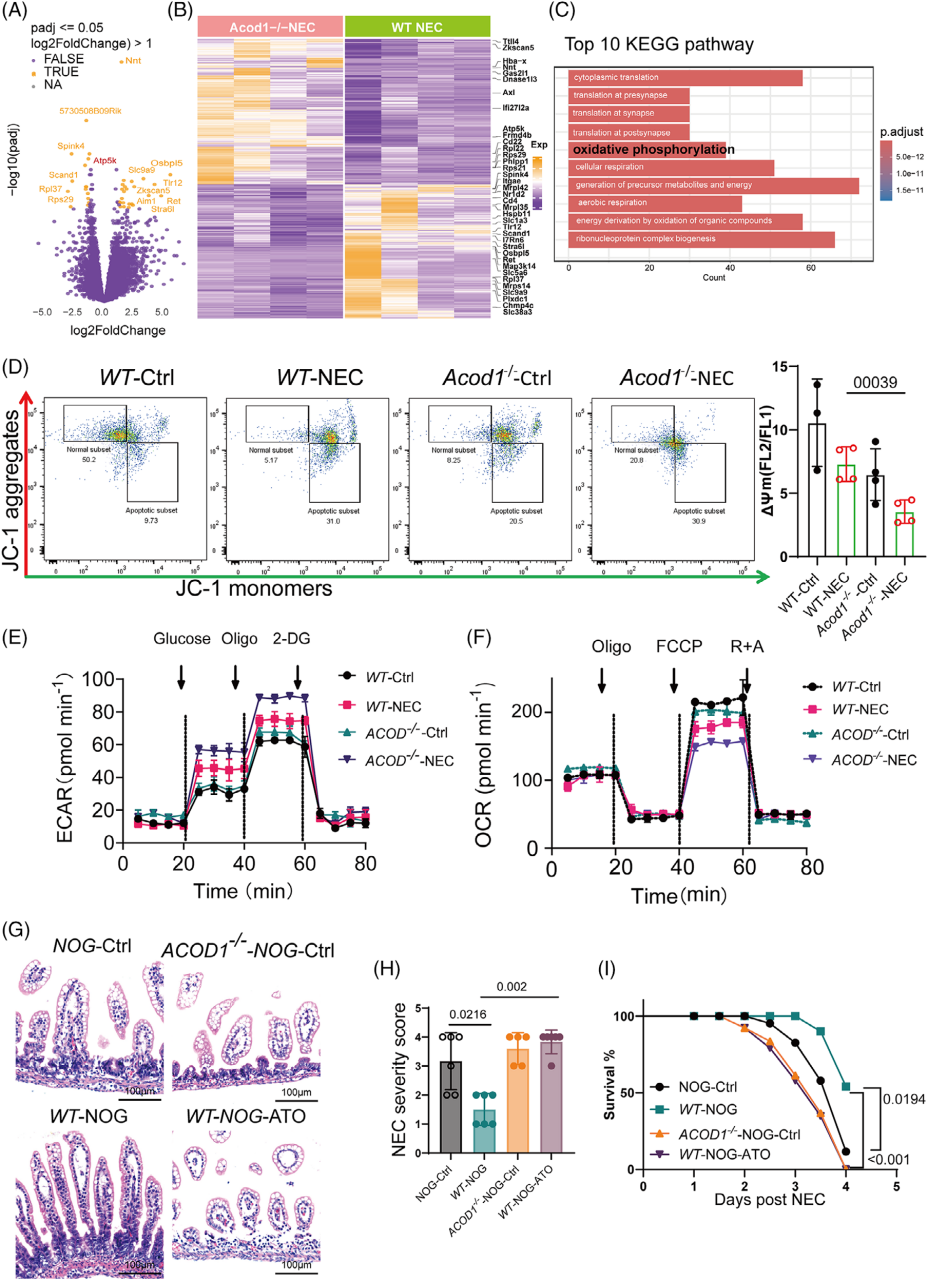
Deficiency promotes macrophages switch towards glycolysis in necrotizing enterocolitis (NEC). (A) Volcano plot and heatmap (B) display of differential genes in Smart‐RNA‐seq analysis. (C) Top 10 enriched pathways in KEGG pathway enrichment analysis. (D) JC‐1 staining was determined by flow cytometry, MMP = Red/Green mean ratio, *n* = 4 per group. (E) OCR profile plot and (F) ECAR profile plot in macrophage (day 3 of cell culture) from *WT* and *ACOD1* ^−/−^ mice, determined by mitochondrial stress test assay (*n *= 16 per group). (G) Representative images of H&E‐stained of the intestinal epithelial tissue. (H) Severity score and survival curve (I) of *WT* and *ACOD1^−/−^* mice, NOG‐Ctrl (n = 6), *WT*‐NOG (*n* = 6), *ACOD1^−/−^*‐NOG‐Ctrl (*n* = 5), *WT*‐NOG (*n* = 5). Data represent mean ± SD, Wilcoxon signed‐rank test, Two‐tailed (D and H), Log‐rank test (I). *p*‐value as shown in Figure.

We performed Smart‐RNA‐sequencing of macrophages from NEC *WT* and *ACOD1^−/−^* mice to create a differential gene expression matrix in order to examine how *ACOD1* deficiency enhances proinflammatory polarization in NEC (Figure 5A,B). We identified a significant enrichment of differentially expressed genes in the OXPHOS pathway, as revealed by gene ontology (GO) enrichment (Figure S3A,B and E). Kyoto Encyclopedia of Genes and Genomes (KEGG) enrichment analysis further revealed that these genes in WT and *ACOD1^−/−^* macrophages are primarily involved in mitochondrial functions, including OXPHOS, generation of precursor metabolism and energy and energy derivation by organic compounds (Figure 5C, Figure S3A,B and E). These findings indicate that the differences in macrophage differentiation resulting from *ACOD1* deficiency may be mediated by mitochondrial function, especially the modulation of cellular OXPHOS. As master regulators of metabolism, mitochondria play crucial roles in the function of macrophages. Mitochondrial membrane potential (MMP) depolarization is a crucial step in mitochondrial malfunction. Staining of JC‐1 which exists in its monomeric form and emits green fluorescence when MMP drops, was performed to assess the effect of ITA on MMP in macrophages. As shown in Figure 5D, the MMP of macrophages in *ACOD1* ^−/−^ NEC group mice decreased compared with that of WT NEC group mice (Figure 5D). To assess the effect of *ACOD1* deletion on macrophage metabolism in NEC, we performed mitochondrial stress assays. *ACOD1* knockout increased glycolytic activity in NEC macrophages (Figure 5E), while reducing mitochondrial respiration and oxygen consumption (Figure 5F). Quantitative analysis confirmed that *ACOD1* ^−/−^ macrophages exhibited a higher glycolytic capacity than wild‐type controls (62.4 ± 0.5 vs 67.6 ± 1.3 mpH/min, *p* < 0.05). *ACOD1* ^−/−^ macrophages had a greater increase in glycolytic capacity in the NEC group (67.6 ± 1.3 vs. 88.8 ± 0.9 mpH/min, *p* < 0.05) (Table S2). To verify whether the effect of ITA depletion in promoting intestinal inflammatory injury in NEC occurred through mitochondrial function, we injected mitochondrial inhibitor atovaquone (ATO) into the peritoneal cavity of NOG mice after macrophage transplantation to block mitochondrial function. Intestinal pathological damage during NEC was significantly reduced in NOG mice that received *WT* macrophage transplants (Figure 5G). In contrast, ATO‐treated mice showed no significant reduction in intestinal damage, with disease pathology scores being markedly higher than those of the *WT*‐NOG group (Figure 5H). Furthermore, survival rates in the ATO intervention group were markedly worse to those in the *WT*‐NOG cohort (Figure 5I). These results suggest that *ACOD1* deficiency promotes NEC by enhancing mitochondrial function in macrophages.

### 2.6 4OI plays a therapeutic role in NEC by promoting mitochondrial activity

**FIGURE 6:**
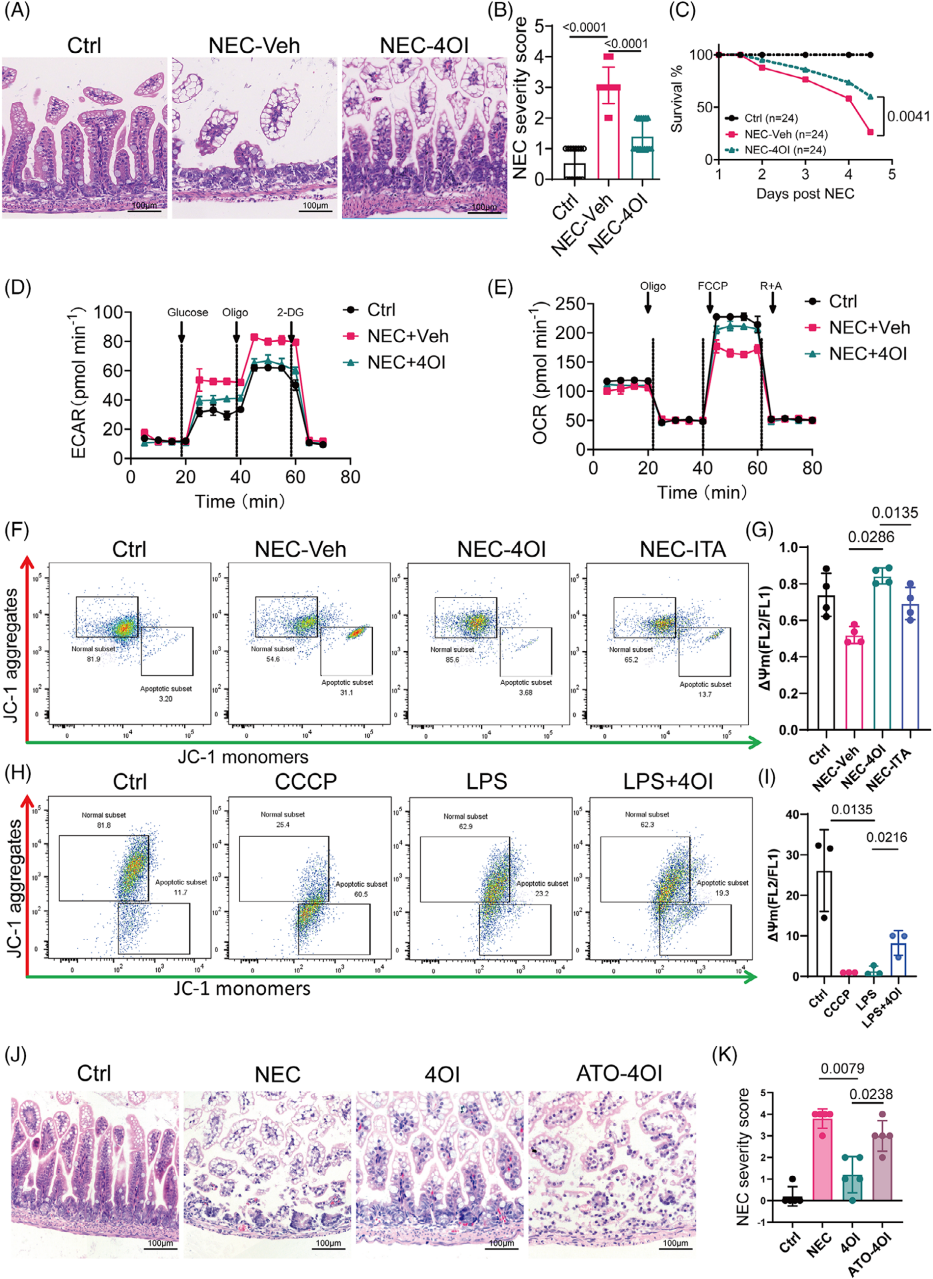
4OI plays a therapeutic role in necrotizing enterocolitis (NEC) by promoting mitochondrial activity. (A) Representative images of H&E‐stained of intestinal epithelial tissue of mice with indicated treatment. (B) Severity score. (C) survival curve. Ctrl, NEC‐Vehicle, NEC‐4OI (*n* = 24 per group). (D) OCR profile plot and (E) ECAR profile plot in macrophage (day 3 of cell culture) from NEC mice treated with 4OI compared with control group, determined by mitochondrial stress test assay. Ctrl (*n *= 4), NEC‐Veh (*n *= 6), NEC‐4OI (*n* = 5). (F) JC‐1 staining in macrophage was determined by flow cytometry. Ctrl, NEC‐Veh, NEC‐4OI, NEC‐ITA, (*n* = 4 per group). (G) quantitative statistical analysis of MMP, MMP = Red/Green mean ratio. (H) JC‐1 staining in THP‐1 cell was determined by flow cytometry, Ctrl, CCCP, LPS, LPS‐4OI (*n* = 3 per group). (I) Quantitative statistical analysis of MMP in THP‐1 cell, MMP = Red/Green mean ratio. (J) Representative images of H&E‐stained intestinal epithelial tissue of NOG mice with treatment of macrophage from ACOD1‐/‐ mice or WT mice macrophage and indicated treatment. ATO, atovaquone. (K) Severity score of NOG mice with indicated treatment. Ctrl, NEC, 4OI, NEC‐4OI, (*n* = 15 per group). Data represent mean ± SD, Unpaired *t*‐test, Two‐tailed (B); Wilcoxon signed‐rank test, Two‐tailed (G I K), Log‐rank test, *n* = 24 per group (C). *p*‐value as shown in Figure.

We evaluated ITA's role in NEC by intraperitoneally administering 4OI, a membrane‐permeable ITA mimic, for 4d alongside NEC induction. H&E staining of the intestinal epithelial tissue showed that 4OI supplementation effectively reduced typical intestinal damage (Figure 6A) and significantly lowered the pathological damage score during NEC (Figure 6B). Additionally, 4OI treatment significantly improved the survival rate of NEC mice (Figure 6C). Furthermore, 4OI treatment reduced macrophage infiltration and the expression of *IL‐6* and *CD68* (Figure S4D,E). Subsequently, we aimed to confirm the possible control of macrophage metabolism by 4OI in NEC. Consistent with the previous findings, the reduced extracellular acidification rate (ECAR) indicated that 4OI supplementation reduced the reliance of macrophages on glycolysis (Figure 6D). Furthermore, compared with vehicle‐treated NEC animals, 4OI‐treated mice showed a substantial improvement in mitochondrial respiratory capacity of macrophages (Figure 6E). Quantitative analysis confirmed that glycolytic capacity was suppressed in NEC mice treated with 4OI compared with the controls (64.8 ± 0.4 vs. 80.5 ± 0.2 mpH/min, *p* < 0.001) (Table S3). As shown in Figure 6F, the MMP of macrophages from the 4OI treated NEC mice was higher than that of the positive control group (NEC‐vehicle group). Furthermore, the specific marker CD64 in M1 macrophages in the intestine tissues of the 4OI treatment cohort mice was significantly downregulated (Figure S4G). However, no significant change in free ROS (Figure S4F). We conducted in vitro using THP‐1 cells treated with CCCP as a positive control group. The results showed that LPS also induced a decline in mitochondrial function in cells (Figure 6H). Meanwhile, 4OI treatment effectively inhibited the LPS‐induced reduction in MMP (Figure 6H). The increase in superoxide level in the mitochondria is an important indicator of mitochondrial dysfunction. The fluorescence of MitoSOX Red, a fluorescent probe for imaging superoxide radicals in the mitochondria of living cells, weakened upon 4OI treatment in THP1 cells (Figure S4I). To further validate the therapeutic role of ITA in NEC by promoting mitochondrial activity, we used macrophages from *ACOD1* ^−/−^ mice after ATO intervention and transplanted them into NOG mice, which were intraperitoneally injected with 4OI. The experimental results showed that in the NOG mouse model of *ACOD1* ^−/−^ mice, 4OI treatment effectively alleviated NEC‐induced intestinal mucosal injury. However, 4OI failed to significantly protect against NEC‐induced intestinal mucosal injury in a mouse model where ATO inhibited mitochondrial activity. (Figure 6J,K). Since 4OI is not a natural product, to evaluate its in vivo toxicity, we administered it via intraperitoneal injection at a dose of 40 mg/kg/d for 3 consecutive days. On the 4th day, the mice were euthanized, and the liver and kidneys were harvested. H&E staining revealed no apparent liver or kidney lesions. (Figure S5). In addition, we administered ITA to NEC mice via intraperitoneal injection and H&E staining indicated that pathological damage to the intestinal epithelial tissue in NEC mice was significantly mitigated after ITA intervention, accompanied by a reduction in the pathological severity score (Figure S4A,B). Furthermore, in contrast to that of the solvent injected group, the survival curve of the ITA‐treated group showed an upward trend (Figure S4C). In conclusion, 4OI supplementation improves NEC by reversing the macrophage redox balance.

## 3 DISCUSSION

*ACOD1* is the key enzyme responsible for synthesizing ITA and plays broader roles in immune regulation, such as the modulation of mitochondrial function, redox homeostasis, and inflammasome activity. Current evidence strongly indicates that the immune regulatory function of *ACOD1* is primarily driven by ITA synthesis. Our results suggest that ITA is depleted during NEC progression due to a decrease in its synthetic precursor. This finding aligns with those of previous studies indicating that Acod1 expression is elevated under inflammatory conditions.^23^ Because *ACOD1* expression is the sole regulator of ITA synthesis, we used an *ACOD1* knockout mouse model to mimic the in vivo environment of ITA depletion. Using *ACOD1*‐deficient mice, we showed that the lack of *ACOD1* exacerbates intestinal damage and inflammation in NEC, characterized by increased pro‐inflammatory cytokine production and damage to the intestinal lamina propria.

An imbalance between pro‐inflammatory and anti‐inflammatory signals is a significant contributor to the cascade of inflammation during intestinal inflammatory damage. Elevated levels of pro‐inflammatory mediators, including TLR4, NF‐κB, TNF, PAF, IL‐18, interferon‐γ, IL‐6, IL‐8 and IL‐1β, lead to sustained intestinal damage. Simultaneously, defects in anti‐inflammatory regulatory mechanisms, such as those involving IL‐1Ra, TLR9, PAF lyase, TGF‐β1 and 2, IL‐10, and regulatory T cells reinforce the pro‐inflammatory environment.^24^ This imbalance triggers an amplified immune response, which not only disrupts local intestinal homeostasis but also exacerbates the condition by inducing systemic metabolic alterations.^25^

In NEC, the inflammatory cascade resulting from a reduction in immunosuppressive cells and an increase in pro‐inflammatory signals is a significant contributor to intestinal barrier impairment. Our previous research demonstrated that both the quantity and functionality of MDSCs within the immunosuppressive cell population were markedly diminished in preterm infants with NEC compared with their term counterparts.^26^ As shown in this research, ITA deficiency amplified the inflammatory cascade in NEC models. But where do these increased levels of inflammatory cytokines come from?

The results of this study indicate that ITA deletion not only promotes the transformation of macrophages to the pro‐inflammatory M1 phenotype but also leads to a significant shift in the metabolic phenotype of macrophages towards glycolysis. Macrophages exhibit metabolic plasticity.^27^ Among them, pro‐inflammatory M1 macrophages mainly rely on glycolysis to provide energy, and their metabolic characteristics are characterized by TCA cycle disruption and mitochondrial dysfunction.^10^ M1 macrophages express low levels of mitochondrial genes (e.g., PPARγ), high levels of glycolytic enzymes (e.g., HK2 and LDHA), and pro‐inflammatory factors (e.g., HIF‐1α and NF‐κB). Their metabolism and immune functions are regulated by TCA cycle enzymes.^11^ Previous studies have shown that ITA can effectively inhibit LPS‐induced elevation of ROS levels and ECAR by activating the anti‐inflammatory program through Nrf2 activation and SDH inhibition.^28^ Upon inflammatory stimuli, ITA modifies the Cys 22 residue of GAPDH with a similar dipropylene glycol moiety, inhibiting glycolysis.^29^ In the present study, gene expression analyses revealed downregulation of genes involved in OXPHOS and mitochondrial function in *ACOD1*‐deficient macrophages. In contrast, M2 macrophages depend on the TCA cycle and OXPHOS. Thus, macrophage differentiation is thus regulated by metabolic reprogramming balance.^11^ Our results indicate that 4OI, a derivative of ITA, influences the metabolic phenotype of macrophages and effectively alleviates intestinal inflammatory damage in NEC. This suggests that 4OI can be used to treat NEC by regulating the metabolism of macrophages, and that targeting the *ACOD1*‐ITA axis offers a novel therapeutic strategy for NEC.

Macrophages are central players in the innate immune response and are highly plastic cells that play dual roles in regulation of immune responses and immune tolerance in NEC.^6^, ^26^ Skewing towards the M1 phenotype contributes to the maintenance of inflammation and tissue damage in NEC.

Metabolic reprogramming is a critical aspect of macrophage function and polarization.^30^ OXPHOS is the main biochemical pathway through which cells generate adenosine triphosphate (ATP) via located on the inner mitochondrial membrane and the electrochemical gradient. Damage to the mitochondrial electron transport chain leads to increased electron leakage, resulting in excessive ROS production and decrease in MMP levels.^31^ This damage reduces the efficiency of OXPHOS and forces cells to switch to the glycolytic pathway to rapidly generate ATP. Macrophage differentiation is metabolism dependent, and M1 macrophages rely on glycolysis for energy production.^27^

Our research findings indicate that ITA‐deficient mice, the MMP levels of macrophages exhibited a significant decrease, accompanied by enhanced glycolysis. Mitochondrial dysfunction induced by the absence of ITA promotes the transformation of macrophages to a pro‐inflammatory phenotype and further aggravates the inflammatory cascade reaction in NEC. Additionally, the increased ROS production observed in these macrophages may result from mitochondrial dysfunction further exacerbating tissue damage.^32^

Prior studies have shown that ITA inhibits mitochondrial respiratory chain complex II activity and diminishes mitochondrial respiratory capacity by suppressing SDH.^33^ However, our study revealed that 4OI intervention significantly recovered the mitochondrial function of macrophages. This phenomenon may be explained by following reasons. First, low‐dose ITA may enhance the cell's antioxidant capacity by activating the Nrf2‐KEAP1 pathway, thereby alleviating ROS‐induced mitochondrial damage.^28^ Second, it may activate the transcription factor TFEB to clear damaged mitochondria and enhance the functionality of the remaining mitochondria.^20^

The therapeutic potential of 4OI in NEC was a significant finding in the present study. Treatment with 4OI reduced intestinal damage, decreased pro‐inflammatory cytokine levels, and improved survival rates in NEC mice. 4OI is a cell‐permeable derivative of ITA that mimics anti‐inflammatory effects.^16^ Previous studies have indirectly reported the distribution of 4OI in multiple organs in mice following intraperitoneal injection using pharmacodynamic experiments.^34^, ^35^, ^36^, ^37^, ^38^ Using proteomics, Liao et al. confirmed that 4OI targeted GAPDH protein, which is widely present in all organs after intraperitoneal injection, suggesting its multiorgan bioavailability.^39^ Our data suggest that 4OI treatment restores mitochondrial function and redox balance in macrophages, as evidenced by increased mitochondrial respiration and decreased glycolytic reliance. Furthermore, 4OI treatment reduced ROS production, which is crucial in preventing oxidative stress‐induced cellular damage. Excessive ROS levels not only disrupt the electron transport chain but may also damage the mitochondrial DNA, resulting in a compromised cellular energy supply.^40^, ^41^

ATO is a mitochondrial function inhibitor. It blocks electron transfer by inhibiting complex III of the mitochondrial electron transport chain thereby inhibiting ATP synthesis. It is an important tool for studying mitochondrial dysfunction and energy metabolism disorders. After intervention with ATO, macrophages were unable to perform their functions in NEC mice, an outcome consistent with that of macrophages lacking *ACOD1*. The importance of mitochondrial function in macrophage‐mediated inflammation is highlighted by our findings using the mitochondrial inhibitor ATO.^42^ Inhibition of mitochondrial activity abrogated the protective effects of macrophage transplantation and 4OI treatment in NEC models. This suggests that the beneficial effects of 4OI are partly mediated through the preservation of mitochondrial function and promotion of OXPHOS in macrophages. Mitochondrial integrity is essential for regulating macrophage polarization and controlling inflammatory responses. Therefore, interventions that support mitochondrial function may have therapeutic benefits in inflammatory diseases such as NEC.

Despite the findings, our study has several limitations that should be considered. First, although we demonstrated the protective role of 4‐OI in mouse models of NEC, the translation of these findings to human patients should be further investigated. Although 4‐OI mimics the anti‐inflammatory effects of ITA, Swain et al. highlighted functional differences,^16^ therefore, future clinical studies should prioritize the use of unmodified ITA and optimize its poor membrane permeability. Second, the exact mechanisms by which ITA modulates mitochondrial function and macrophage metabolism remain unclear. Further research is necessary to elucidate the signalling pathways involved and to identify potential targets for therapeutic intervention. Lastly, NEC is a complex disease impacted by both hereditary and environmental factors. While our study focused on the role of macrophages and ITA, other cell types and pathways may also contribute to disease progression and should be explored in future studies.

## 4 CONCLUSION

Our findings underscore the critical role of Acod1‐mediated ITA production in regulation of macrophage metabolism and function in NEC. ITA deficiency leads to enhanced pro‐inflammatory macrophage polarization, increased glycolysis, mitochondrial dysfunction, and increased ROS production, all of which contribute to intestinal inflammation and damage. Supplementation with the ITA derivative 4OI mitigated these effects via rescuing macrophage mitochondrial respiratory function, highlighting its therapeutic potential. For clinical translation, unmodified ITA should be considered as the preferred candidate owing to its well‐documented immuno‐regulatory properties. Nonetheless, 4OI can serve as a valuable compound during the proof‐of‐concept phase, providing essential support for preliminary investigations.

## 5 MATERIALS AND METHODS

### 5.1 Human participants

The included participants were neonates diagnosed via imaging examinations and met the clinical diagnostic criteria for NEC. Specifically, they included neonates with abdominal radiographs showing intestinal wall gas accumulation or portal vein gas accumulation or those diagnosed by surgery.

The exclusion criteria were as follows: severe congenital diseases such as complex congenital heart disease or chromosomal abnormalities; other intestinal diseases such as congenital intestinal atresia or intestinal malrotation; receipt of immunomodulatory treatment immediately after birth; or sepsis or other severe systemic infections for other reasons. Clinical plasma samples were obtained from the Department of Hematology at Guangzhou Women and Children's Medical Center, and these included peripheral blood samples from 14 patients diagnosed with NEC and a control group consisting of 7 premature infants and neonatal dyspnoea patients (NC). Tissue samples were collected from the Guangdong Women and Children's Medical Center. All participants provided written informed consent before sample collection (K‐2022‐030‐01). All NEC patients were diagnosed according to the standard diagnostic criteria for neonatal acute enterocolitis. According to the widely used BELL scale in clinical practice, the included cases were graded based on clinical manifestations, imaging examinations, and laboratory tests.^43^ The detailed clinical information is provided in Table S1.

### 5.2 Animals

C57BL/6 mice were obtained from Zhuhai BesTest Biotechnology Co., Ltd. (China), while *ACOD1* ^−/−^ mice were provided by the Yun Zhao's team at the Department of General Surgery, BenQ Medical Center, Affiliated BenQ Hospital of Nanjing Medical University. Myeloid‐specific *ACOD1*‐knockout (*ACOD11^fl/fl^LysM^cre^*) mice were generated by crossing *ACOD1^fl/fl^* mice (S‐CKO‐03141) with LysM‐Cre mice (004781) purchased from Cyagen Biosciences and Jackson Laboratory (ME, USA), respectively. Floxed littermates (*ACOD1^fl/fl^*) were used as controls for *ACOD1^fl/fl^LysM^cre^* mice. All animal experiments were approved by the Institutional Animal Care and Use Committee (the approval number: 202312005).

### 5.3 Construction of a neonatal NEC mouse model

Seven‐day‐old C57BL/6 pups were divided into two groups: control group (Ctrl) and experimental (NEC). The NC group pups were naturally nurtured by their mothers, whereas the NEC pups were gavaged daily with 30 mg/kg of lipopolysaccharide (LPS, Sigma‐Aldrich) using a clean peripherally inserted central catheter. They were then fed a formula consisting of Similac Advance infant formula (Abbott Nutrition) and Esbilac puppy milk replacer (PetAg) in a 2:1 ratio.^44^ Subsequently, the pups were exposed to 5% oxygen and 95% nitrogen for 10 min to induce asphyxia, followed by cold stress at 4°C for 10 min. This procedure was conducted twice daily for 3 consecutive days. For exogenous ITA administration experiments, mice were intraperitoneally injected with 40 mg/kg 4OI, Selleckchem, Cat No. S5929) or ITA (Cat No. SS3095; Selleckchem) from the day before NEC modelling until the third day of modelling.

### 5.4 Cell culture

THP‐1 cells were obtained from the Cell Bank of Chinese Academy of Sciences. They were cultured in Roswell Park Memorial Institute 1640 Medium (Gibco/Thermo Fisher Scientific) with 10% fetal bovine serum (Gibco) at 37°C in a humidified incubator with 5% CO_2_.

### 5.5 Cell transplantation

Macrophages were isolated from the spleens of wild‐type C57BL/6 or Acod1^−/−^ mice, sorted, and intraperitoneally injected into NOG (NOD/Shi‐scid/IL‐2Rγnull) immunodeficient mice (10^6^ cells per mouse) on days 1 and 3 of NEC induction. The mice were sacrificed 4d post‐induction and samples were collected for subsequent analyses.^45^

### 5.6 Targeted metabolomics

Within 15 min of collecting peripheral blood in ethylenediaminetetraacetic acid anticoagulating tubes, the plasma was separated via centrifugation (1500 × g, 4°C, 10 min) for TCA cycle metabolites detection. Targeted metabolomic analysis was performed on a Thermo Scientific™ TSQ Altis™ triple quadrupole mass spectrometer coupled with a Thermo Scientific Vanquish™ Flex ultra‐high‐performance liquid chromatography (UHPLC) system to quantify 13 organic acids associated with the TCA cycle. Standards for the 13 organic acids were obtained from Shanghai Zhenzhun Biotechnology Co., Ltd. Methanol, acetonitrile, and formic acid (all LC‐MS grade; Thermo Fisher Scientific), and ultrapure water (Mill‐Q; Millipore), were used as reagents.

### 5.7 Flow cytometry

For intracellular cytokine staining, the cell suspension was stimulated using a Cell Stimulation Kit at 37°C in a 5% CO₂ for 4 h. To minimize nonspecific binding, cells were pre‐treated with Fc Block CD16/CD32 antibodies (Cat No. 14‐0161‐81, Invitrogen). Specific surface molecule antibodies were carefully chosen and used to stain the cells at a 1:100 dilution for 30 min at 4°C. After staining for surface markers, the cells were fixed and permeabilized using a Foxp3 Cytofix/Cytoperm kit (Cat No. 554714, Tonbo Biosciences). Finally, intracellular and nuclear staining was performed at 4°C with the appropriate antibodies, following the manufacturer's instructions. Details of the antibodies used are provided in the key resource table (Supplementary Materials). Additionally, cells were incubated with Mito‐Green (Cat No. C1048, Beyotime) at 37°C for 30 min to evaluate mitochondrial function. All flow cytometry data were collected on a BD LSRFortessa‐X20 instrument (BD Biosciences) and subsequently analysed using FlowJo software (version 10; FlowJo LLC).

### 5.8 Fluorescence‐activated cell sorting

After preparing the cell suspensions from the tissues, cells were pre‐treated with Fc Block CD16/CD32 antibodies. The cells were then stained with specific surface molecule antibodies, carefully selected for their targets, at a 1:100 dilution for 30 min at 4°C. After staining, the cells were resuspended in flow cytometry tubes and analysed using a BD FACSAria™ III flow cytometer (BD Biosciences). Specific information regarding the antibodies used for flow cytometry is provided in key resources table (Supplementary Materials).

### 5.9 Single cell RNA sequencing

The raw sequence data reported in this paper have been deposited in the Genome Sequence Archive of the BIG Data Center, Chinese Academy of Sciences under the accession code CRA024721 and are publicly accessible at http://bigd.big.ac.cn/gsa. Single‐cell RNA sequencing (scRNA‐seq) data were obtained from resected intestinal tissues of *ACOD1^−/−^* and *WT* NEC mice. First, individual cells were encapsulated in gel beads containing barcodes and primers using 10x™ GemCode™ Technology to form Gel Bead‐In‐Emulsions (GEMs). mRNA from each cell is reverse transcribed into barcoded cDNA within the GEMs, followed by amplification and purification. Next, sequencing libraries were constructed and sequenced on an Illumina platform (Illumina) to generate sequence data using cell‐specific barcodes. Finally, the data were processed using the Cell Ranger pipeline (v4.0.0) to align the reads and generate gene‐cell count matrices. Cells with fewer than 200 expressed genes or genes detected in fewer than three cells were removed. After merging all samples in Seurat (v4.3.0, R v4.3.1), low‐quality cells were filtered out based on the following criteria: < 1900 UMIs, < 1000 genes per cell, > 30% mitochondrial content, or > 10% erythrocyte marker expression. The Seurat object was normalized and scaled using the LogNormalize and ScaleData functions, respectively. Highly variable genes (*n* = 2,000) were identified using FindVariableGenes, followed by principal component analysis (PCA). Clustering was performed using the top 20 principal components via the graph‐based FindClusters function with a resolution of 0.3. Uniform Manifold Approximation and Projection (UMAP) was used for dimensionality reduction with the RunUMAP function.

Cluster‐specific differentially expressed genes were identified using the Wilcoxon rank‐sum test via FindAllMarkers (parameters: min.pct = 0.25, only.pos = TRUE, logfc.threshold = 0.25, and p.adjust.method = “BH”). Clusters were annotated based on the expression of canonical marker genes.

### 5.10 Statistical analysis

All data are presented as the mean ± standard error of the mean or as individual data points. Statistical analysis between two groups was conducted using an unpaired t‐test or Wilcoxon rank‐sum test based on whether the data were normally distributed. Correlations were analysed using Pearson's correlation analysis. Survival time was analysed using a simple survival analysis (Kaplan–Meier) as implemented in GraphPad Prism (version 9).

More details please refer to Supplementary Materials and methods in Supplementary materials.

## AUTHOR CONTRIBUTIONS

Shuchen Huangfu, Chaoting Lan and Sitao Li wrote the main manuscript text and drafted the work. Shuchen Huangfu and Huijuan Wang contributions to the in vivo experiment and the analysis of data. Chun Yan, Yuling Yang and Yijia Wang contributions to the interpretation of data; Bowen Tian, Yide Mu, Peizhi Zhao and Yan Tian contributions to the acquisition of data; Wei Zhong and Sitao Li revised it critically for important intellectual content; and Limei Zhong, Yongyan Shi and Yufeng Liu contributions to the conception or design of the work and agree to be accountable for all aspects of the work in ensuring that questions related to the accuracy or integrity of any part of the work are appropriately investigated and resolved.

## CONFLICT OF INTEREST STATEMENT

The authors declare no conflicts of interest.

## FUNDING INFORMATION

For Yufeng Liu, National Natural Science Funds (grant no. 82171695); Science and Technology Program of Guangzhou (SL2024A03J01319; SL2024A04J00240). Chaoting Lan is supported by the grant National Natural Science Foundation of China (grant no. 82301955), the Research Foundation of Guangzhou Women and Children's Medical Center for Clinical Doctor (grant no. 2023BS015), the China Postdoctor Science Foundation (grant no. 2023M730791), the Guangdong Basic and Applied Basic Research Foundation (grant no. 2024A1515013190) and the Science and Technology Project of Guangzhou (grant no. 2024A03J1238). Wei Zhong is supported by the grant National Natural Science Foundation of China (grant no. 82370526) and the Science and Technology Project of Guangzhou (grant no. 2024A03J1171). Yan Tian is supported by the Science and Technology Research Project of Education Department of Jiangxi Province (grant no. GJJ2203559) and the Jiangxi Provincial Children's Hospital 2024 First Batch “Qingmiao” Scientific Research Projects (grant no. 2024JXEYQM02). Yongyan Shi is supported by National Natural Science Foundation of China (grant no. 82171709) and the 345 Talent Project of Shengjing Hospital (grant no. M1392).

## ETHICS APPROVAL AND CONSENT TO PARTICIPATE

Ethics approval of this study was granted by the Department of Hematology at Guangzhou Women and Children's Medical Center (approval number: 307B01) The study was conducted in accordance with the Declaration of Helsinki principles.

## CONSENT FOR PUBLICATION

Not applicable.

## Supporting information



Supporting Information

## Data Availability

The data that support the findings of this study are available from the corresponding author upon reasonable request.

## References

[1] Nino DF , Sodhi CP , Hackam DJ . Necrotizing enterocolitis: new insights into pathogenesis and mechanisms. Nat Rev Gastroenterol Hepatol. 2016;13(10):590‐600. doi:10.1038/nrgastro.2016.119 27534694 PMC5124124

[2] Neu J , Walker WA . Necrotizing enterocolitis. N Engl J Med. 2011;364(3):255‐264. doi:10.1056/NEJMra1005408 21247316 PMC3628622

[3] Blakely ML , Tyson JE , Lally KP , et al. Initial Laparotomy versus peritoneal drainage in extremely low birthweight infants with surgical necrotizing enterocolitis or isolated intestinal perforation: a multicenter randomized clinical trial. Ann Surg. 2021;274(4):e370‐e380. doi:10.1097/SLA.0000000000005099 34506326 PMC8439547

[4] Cao X , Zhang L , Jiang S , et al. Epidemiology of necrotizing enterocolitis in preterm infants in China: a multicenter cohort study from 2015 to 2018. J Pediatr Surg. 2022;57(3):382‐386. doi:10.1016/j.jpedsurg.2021.05.014 34175121

[5] Cho SX , Rudloff I , Lao JC , et al. Characterization of the pathoimmunology of necrotizing enterocolitis reveals novel therapeutic opportunities. Nat Commun. 2020;11(1):5794. doi:10.1038/s41467-020-19400-w 33188181 PMC7666196

[6] Wei J , Meng Z , Li Z , Dang D , Wu H . New insights into intestinal macrophages in necrotizing enterocolitis: the multi‐functional role and promising therapeutic application. Front Immunol. 2023;14:1261010. doi:10.3389/fimmu.2023.1261010 37841247 PMC10568316

[7] Kim JE , Li B , Fei L , et al. Gut microbiota promotes stem cell differentiation through macrophage and mesenchymal niches in early postnatal development. Immunity. 2022;55(12):2300‐2317. doi:10.1016/j.immuni.2022.11.003 36473468

[8] Faas M , Ipseiz N , Ackermann J , et al. IL‐33‐induced metabolic reprogramming controls the differentiation of alternatively activated macrophages and the resolution of inflammation. Immunity. 2021;54(11):2531‐2546. doi:10.1016/j.immuni.2021.09.010 34644537 PMC7617137

[9] Jia N , Gao Y , Li M , et al. Metabolic reprogramming of proinflammatory macrophages by target delivered roburic acid effectively ameliorates rheumatoid arthritis symptoms. Signal Transduct Target Ther. 2023;8(1):280. doi:10.1038/s41392-023-01499-0 37500654 PMC10374631

[10] Pan X , Zhu Q , Pan LL , Sun J . Macrophage immunometabolism in inflammatory bowel diseases: from pathogenesis to therapy. Pharmacol Ther. 2022;238:108176. doi:10.1016/j.pharmthera.2022.108176 35346728

[11] Li M , Yang Y , Xiong L , Jiang P , Wang J , Li C . Metabolism, metabolites, and macrophages in cancer. J Hematol Oncol. 2023;16(1):80. doi:10.1186/s13045-023-01478-6 37491279 PMC10367370

[12] Michelucci A , Cordes T , Ghelfi J , et al. Immune‐responsive gene 1 protein links metabolism to immunity by catalyzing itaconic acid production. Proc Natl Acad Sci U S A. 2013;110(19):7820‐7825. doi:10.1073/pnas.1218599110 23610393 PMC3651434

[13] Peace CG , O'Neill LA . The role of itaconate in host defense and inflammation. J Clin Invest. 2022;132(2):e148548. doi:10.1172/JCI148548 35040439 PMC8759771

[14] Wu R , Chen F , Wang N , Tang D , Kang R . ACOD1 in immunometabolism and disease. Cell Mol Immunol. 2020;17(8):822‐833. doi:10.1038/s41423-020-0489-5 32601305 PMC7395145

[15] Liu C , Fu C , Sun Y , et al. Itaconic acid regulation of TFEB‐mediated autophagy flux alleviates hyperoxia‐induced bronchopulmonary dysplasia. Redox Biol. 2024;72:103115. doi:10.1016/j.redox.2024.103115 38554522 PMC10998238

[16] Swain A , Bambouskova M , Kim H , et al. Comparative evaluation of itaconate and its derivatives reveals divergent inflammasome and type I interferon regulation in macrophages. Nat Metab. 2020;2(7):594‐602. doi:10.1038/s42255-020-0210-0 32694786 PMC7378276

[17] Cordes T , Lucas A , Divakaruni AS , Murphy AN , Cabrales P , Metallo CM . Itaconate modulates tricarboxylic acid and redox metabolism to mitigate reperfusion injury. Mol Metab. 2020;32:122‐135. doi:10.1016/j.molmet.2019.11.019 32029222 PMC6961711

[18] Tomlinson KL , Riquelme SA , Baskota SU , et al. Staphylococcus aureus stimulates neutrophil itaconate production that suppresses the oxidative burst. Cell Rep. 2023;42(2):112064. doi:10.1016/j.celrep.2023.112064 36724077 PMC10387506

[19] Ye D , Wang P , Chen LL , Guan KL , Xiong Y . Itaconate in host inflammation and defense. Trends Endocrinol Metab. 2024;35(7):586‐606. doi:10.1016/j.tem.2024.02.004 38448252

[20] Chen B , Liu Y , Luo S , et al. Itaconic acid ameliorates necrotizing enterocolitis through the TFEB‐mediated autophagy‐lysosomal pathway. Free Radic Biol Med. 2025;226:251‐265. doi:10.1016/j.freeradbiomed.2024.11.035 39571950

[21] Du L , Lin L , Li Q , et al. IGF‐2 preprograms maturing macrophages to acquire oxidative phosphorylation‐dependent anti‐inflammatory properties. Cell Metab. 2019;29(6):1363‐1375. doi:10.1016/j.cmet.2019.01.006 30745181

[22] Gershner GH , Hunter CJ . Redox chemistry: implications for necrotizing enterocolitis. Int J Mol Sci. 2024;25(15):8416. doi:10.3390/ijms25158416 39125983 PMC11312856

[23] Ryan DG , O'Neill L . Krebs cycle reborn in macrophage immunometabolism. Annu Rev Immunol. 2020;38:289‐313. doi:10.1146/annurev-immunol-081619-104850 31986069

[24] Neurath MF . Strategies for targeting cytokines in inflammatory bowel disease. Nat Rev Immunol. 2024;24(8):559‐576. doi:10.1038/s41577-024-01008-6 38486124

[25] Sampah M , Hackam DJ . Dysregulated mucosal immunity and associated pathogeneses in preterm neonates. Front Immunol. 2020;11:899. doi:10.3389/fimmu.2020.00899 32499778 PMC7243348

[26] He YM , Li X , Perego M , et al. Transitory presence of myeloid‐derived suppressor cells in neonates is critical for control of inflammation. Nat Med. 2018;24(2):224‐231. doi:10.1038/nm.4467 29334374 PMC5803434

[27] Wang S , Liu G , Li Y , Pan Y . Metabolic Reprogramming induces macrophage polarization in the tumor microenvironment. Front Immunol. 2022;13:840029. doi:10.3389/fimmu.2022.840029 35874739 PMC9302576

[28] Mills EL , Ryan DG , Prag HA , et al. Itaconate is an anti‐inflammatory metabolite that activates Nrf2 via alkylation of KEAP1. Nature. 2018;556(7699):113‐117. doi:10.1038/nature25986 29590092 PMC6047741

[29] Yang W , Wang Y , Huang Y , et al. 4‐Octyl itaconate inhibits aerobic glycolysis by targeting GAPDH to promote cuproptosis in colorectal cancer. Biomed Pharmacother. 2023;159:114301. doi:10.1016/j.biopha.2023.114301 36706634

[30] O'Neill LA , Pearce EJ . Immunometabolism governs dendritic cell and macrophage function. J Exp Med. 2016;213(1):15‐23. doi:10.1084/jem.20151570 26694970 PMC4710204

[31] Nolfi‐Donegan D , Braganza A , Shiva S . Mitochondrial electron transport chain: oxidative phosphorylation, oxidant production, and methods of measurement. Redox Biol. 2020;37:101674. doi:10.1016/j.redox.2020.101674 32811789 PMC7767752

[32] Song J , Xiao L , Zhang Z , et al. Effects of reactive oxygen species and mitochondrial dysfunction on reproductive aging. Front Cell Dev Biol. 2024;12:1347286. doi:10.3389/fcell.2024.1347286 38465288 PMC10920300

[33] Nemeth B , Doczi J , Csete D , et al. Abolition of mitochondrial substrate‐level phosphorylation by itaconic acid produced by LPS‐induced Irg1 expression in cells of murine macrophage lineage. Faseb J. 2016;30(1):286‐300. doi:10.1096/fj.15-279398 26358042

[34] Kachler K , Andreev D , Thapa S , et al. Acod1‐mediated inhibition of aerobic glycolysis suppresses osteoclast differentiation and attenuates bone erosion in arthritis. Ann Rheum Dis. 2024;83(12):1691‐1706. doi:10.1136/ard-2023-224774 38964754 PMC11671873

[35] Kim HW , Yu AR , Lee JW , et al. Aconitate Decarboxylase 1 deficiency exacerbates mouse colitis induced by dextran sodium sulfate. Int J Mol Sci. 2022;23(8):4392. doi:10.3390/ijms23084392 35457208 PMC9025264

[36] Yang Y , Li Y , Yang W , et al. Protecting effects of 4‐octyl itaconate on neonatal hypoxic‐ischemic encephalopathy via Nrf2 pathway in astrocytes. J Neuroinflammation. 2024;21(1):132. doi:10.1186/s12974-024-03121-8 38760862 PMC11102208

[37] Zhao N , Yi M , Zhang LJ , Zhang QX , Yang L . 4‐Octyl Itaconate Attenuates neuroinflammation in experimental autoimmune encephalomyelitis via regulating microglia. Inflammation. 2025;48(1):151‐164. doi:10.1007/s10753-024-02050-1 38761250

[38] Yang J , Duan C , Wang P , et al. 4‐Octyl Itaconate Alleviates myocardial ischemia‐reperfusion injury through promoting angiogenesis via ERK signaling activation. Adv Sci (Weinh). 2025;12(10):e2411554. doi:10.1002/advs.202411554 39836624 PMC11904966

[39] Liao ST , Han C , Xu DQ , Fu XW , Wang JS , Kong LY . 4‐Octyl itaconate inhibits aerobic glycolysis by targeting GAPDH to exert anti‐inflammatory effects. Nat Commun. 2019;10(1):5091. doi:10.1038/s41467-019-13078-5 31704924 PMC6841710

[40] Casati SR , Cervia D , Roux‐Biejat P , Moscheni C , Perrotta C , De Palma C . Mitochondria and reactive oxygen species: the therapeutic balance of powers for duchenne muscular dystrophy. Cells. 2024;13(7):574. doi:10.3390/cells13070574 38607013 PMC11011272

[41] Addabbo F , Montagnani M , Goligorsky MS . Mitochondria and reactive oxygen species. Hypertension. 2009;53(6):885‐892. doi:10.1161/HYPERTENSIONAHA.109.130054 19398655 PMC2716801

[42] Skwarski M , McGowan DR , Belcher E , et al. Mitochondrial inhibitor atovaquone increases tumor oxygenation and inhibits hypoxic gene expression in patients with non‐small cell lung cancer. Clin Cancer Res. 2021;27(9):2459‐2469. doi:10.1158/1078-0432.CCR-20-4128 33597271 PMC7611473

[43] Bell MJ , Ternberg JL , Feigin RD , et al. Neonatal necrotizing enterocolitis. Therapeutic decisions based upon clinical staging. Ann Surg. 1978;187(1):1‐7. doi:10.1097/00000658-197801000-00001 413500 PMC1396409

[44] Luo S , Zeng Y , Chen B , et al. Vitamin E and GPX4 cooperatively protect treg cells from ferroptosis and alleviate intestinal inflammatory damage in necrotizing enterocolitis. Redox Biol. 2024;75:103303. doi:10.1016/j.redox.2024.103303 39137584 PMC11372871

[45] Wan X , Xie B , Sun H , et al. Exosomes derived from M2 type tumor‐associated macrophages promote osimertinib resistance in non‐small cell lung cancer through MSTRG.292666.16‐miR‐6836‐5p‐MAPK8IP3 axis. Cancer Cell Int. 2022;22(1):83. doi:10.1186/s12935-022-02509-x 35168607 PMC8845243

