# Supplementary material for: Itaconate suppresses neonatal intestinal inflammation via metabolic reprogramming of M1 macrophage
Source: Clin Transl Med. 2025 Jul 17;15(7):e70419. doi: 10.1002/ctm2.70419 (PMC12268792; doi:10.1002/ctm2.70419)
Supplement: Supplementary file 1 — Supporting Information [file CTM2-15-e70419-s001.docx]

**Supplementary materials**

**Supplementary table 1**

**Clinical characteristic of control group and patients with NEC**

| Variables | | Control (n=7)^1^ | NEC-I  (n=3)^1^ | NEC-II  (n=7)^1^ | NEC-III  (n=4)^1^ |
| --- | --- | --- | --- | --- | --- |
| Gender(%) | Male | 4(57.1) | 2(66.6) | 4(57.1) | 2(50.0) |
|  | Female | 3(42.9) | 1(33.3) | 3(42.9) | 2(50.0) |
| gestational age  (mean week) | | 30.384 | 32.9586 | 31.3314 | 31.6629 |
| Birth weight (mean kg) | | 1.61 | 1.71 | 1.36 | 1.54 |
| Multiple pregnancy (%) | | | | | |
| YES | | 42.9 | 66.6 | 28.6 | 60.0 |
| NO | | 57.1 | 33.3 | 71.4 | 40.0 |
| Mode of production (%) | | | | | |
| Cesarean section | | 28.6 | 100 | 28.6 | 100 |
| Natural birth | | 71.4 | 0 | 71.4 | 0 |
| Feeding patterns (%) | | | | | |
| Mixed feeding | | 28.6 | 66.6 | 28.6 | 25.0 |
| Artificial feeding | | 42.9 | 0 | 42.9 | 25.0 |
| Breastfeeding | | 28.6 | 33.3 | 28.6 | 50.0 |
| Apgar score | | | | | |
| 1min | | 7.5 | 8.75 | 8.4 | 8.05 |
| 5min | | 8.9 | 9.5 | 9.15 | 9.3 |
| 10min | | 9.25 | 9.55 | 9.4 | 9.45 |

**Supplementary table 2**

**Statistical Data of the OCR/ECAR Curve in Fig. 5**

| Parameter | WT Ctrl (Mean ± SEM) | WT NEC (Mean ± SEM) | ACOD1-/- Ctrl (Mean ± SEM) | *ACOD1*-/- NEC (Mean ± SEM) | p-value (WT Ctrl vs. ACOD1-/- Ctrl ) | p-value (ACOD1-/- Ctrl vs. ACOD1-/- NEC ) |
| --- | --- | --- | --- | --- | --- | --- |
| Basal OCR (pmol/min)(n=3) | 106.9±1.5 | 103.3±2.2 | 118.1±0.8 | 104.8±0.9 | <0.005 | 0.569 |
| Maximal OCR(n=3) | 216.2±2.2 | 181±3.8 | 201.4±2.9 | 154.3±1 | <0.05 | <0.005 |
| ATP-linked OCR(n=3) | 62.3±3.3 | 52.2±0.6 | 69.1±1.7 | 54.6±0.9 | 0.14 | 0.09 |
| Proton Leak(n=3) | -5.3±1.9 | 1±2.4 | 8.4±2.3 | 2.5±1.1 | <0.05 | 0.6 |
| Spare Respiratory Capacity(n=3) | 109.3±3.7 | 77.7±1.5 | 83.3±3.4 | 49.4±1.7 | <0.05 | <0.001 |
| Basal ECAR (mpH/min)(n=3) | 12.6±0.7 | 11.2±0.4 | 16.7±1.3 | 16.7±1.9 | <0.005 | <0.001 |
| Glucose-induced ECAR(n=3) | 31.4±0.6 | 45.2±3.1 | 33.8±1.4 | 56.5±2 | 0.19 | <0.05 |
| Glycolytic Reserve(n=3) | 31±1.1 | 29.7±3.7 | 33.8±0.1 | 32.3±1.1 | 0.07 | 0.52 |
| Glycolytic Capacity(n=3) | 62.4±0.5 | 74.9±0.7 | 67.6±1.3 | 88.8±0.9 | <0.05 | <0.001 |
| Non-glycolytic Acidification(n=3) | 11.1±0.3 | 13.9±1.5 | 15.1±0.8 | 16.5±0.6 | <0.05 | 0.166 |

Unpaired t-test; Two tailed.

**Supplementary table 3**

**Statistic Data of the OCR/ECAR Curve in Fig. 6**

| Parameter | WT Ctrl (Mean ± SEM) | NEC+Veh (Mean ± SEM) | NEC+4OI (Mean ± SEM) | p-value (WT Ctrl vs. NEC+Veh ) | p-value (NEC+Veh vs. NEC+4OI ) |
| --- | --- | --- | --- | --- | --- |
| Basal OCR (pmol/min)(n=3) | 118.1±0.8 | 104.8±0.9 | 110.5±0.8 | <0.001 | <0.05 |
| Maximal OCR(n=3) | 224.4±0.6 | 169.4±3.9 | 208.8±1.2 | <0.001 | <0.001 |
| ATP-linked OCR(n=3) | 69.1±1.7 | 54.6±0.9 | 61.1±0.4 | <0.005 | <0.005 |
| Proton Leak(n=3) | -2.75±0.25 | -1.6±0.7 | -1±0.8 | 0.18 | 0.6 |
| Spare Respiratory Capacity(n=3) | 106.3±1.2 | 64.6±4.5 | 98.3±1.7 | <0.001 | <0.005 |
| Basal ECAR (mpH/min)(n=5) | 12.6±0.4 | 13.8±0.5 | 11.1±0.3 | 0.1 | <0.005 |
| Glucose-induced ECAR(n=5) | 31.4±0.6 | 52.6±1.5 | 39.6±0. | <0.001 | <0.001 |
| Glycolytic Reserve(n=5) | 30.7±0.8 | 27.9±1.5 | 25.2±0.3 | 0.13 | 0.12 |
| Glycolytic Capacity(n=5) | 62.2±0.5 | 80.5±0.2 | 64.8±0.4 | <0.001 | <0.001 |
| Non-glycolytic Acidification(n=5) | 10.1±0.4 | 11.7±0.6 | 10.6±0.2 | 0.06 | 0.12 |

Unpaired t-test; Two tailed.

**Supplementary table 4**

**Catalog of Primers Employed in the Research**

| **Gene** | **Primer** | **Necleotide Sequence** | **GeneID** |
| --- | --- | --- | --- |
| *Tnf-α* | F | TTCCGAATTCACTGGAGCCTCGAA | 21926 |
|  | R | TGCACCTCAGGGAAGAATCTGGA |  |
| *Il-1β* | F | TTCAGGCAGGCAGTATCACTC | 16176 |
|  | R | GAAGGTCCACGGGAAAGACAC |  |
| *Il-6* | F | AGTTGCCTTCTTGGGACTGA | 3569 |
|  | R | TCCACGATTTCCCAGAGAAC |  |
| *Acod1* | F | CCAAAGAGATTCCACCCTCCC | 16365 |
|  | R | CCTGCGTGGGAAACAGCAAT |  |
| *Cd68* | F | CTTCCCACAGGCAGCACAG | 12514 |
|  | R | AATGATGAGAGGCAGCAAGAGG |  |
| *Cd86* | F | CAGAACTTACGGAAGCACCCA | 12524 |
|  | R | CAGCACGGACTTGAACAACC |  |
| *Cd64* | F | AGGTTCCTCAATGCCAAGTGA | 2209 |
|  | R | GCGACCTCCGAATCTGAAGA |  |
| *Cd206* | F | CTCTGTTCAGCTATTGGACGC | 4630 |
|  | R | CGGAATTTCTGGGATTCAGCTTC |  |
| *Arg1* | F | CTCCAAGCCAAAGTCCTTAGAG | 11846 |
|  | R | AGGAGCTGTCATTAGGGACATC |  |
| *CD86* | F | CTGCTCATCTATACACGGTTACC | 943 |
|  | R | GGAAACGTCGTACAGTTCTGTG |  |
| *CD64* | F | TGGCCTTGAGGTGTCATGC | 14129 |
|  | R | GCAAGAGCAACTTTGTTTCACA |  |
| *CD206* | F | TCCGGGTGCTGTTCTCCTA | 17533 |
|  | R | CCAGTCTGTTTTTGATGGCACT |  |
| *ARG1* | F | GTGGAAACTTGCATGGACAAC | 383 |
|  | R | AATCCTGGCACATCGGGAATC |  |
| *β-actin* | F | CTAGGCACCAGGGTGTGATG | 11461 |
|  | R | GTACATGGCTGGGGTGTTGA |  |
| *GAPHA* | F | GGAGCGAGATCCCTCCAAAAT | 2597 |
|  | R | GGCTGTTGTCATACTTCTCATGG |  |

**Supplementary Materials and methods**

**Induction of macrophage differentiation**

THP1 cells were spread in 6-well plates at 1x10^6^ cells per well. 10μmol/L Phorbol 12-myristate 13-acetate (PMA) was added to the wells and purified for 24h. Cell adhesion was observed under microscope. To induce the polarization of M1-type cells, the activated cells were incubated with 10 pg/ml LPS for 48 hours; Induction of M2-type polarization: Activated cells were incubated with 20 pg/ml IL4 for 48 h, and differentiation efficiency was verified by qPCR.

**ROS scavenge test**

DCFH-DA fluorescent probe was employed for the detection of intracellular reactive oxygen species (ROS). Single-cell suspensions were incubated with 10 μmol/L DCFH-DA reagent at 37°C for 20 minutes, followed by three washes with serum-free medium. The cells were then resuspended in phosphate-buffered saline (PBS) and analyzed using a CytoFLEX flow cytometer coupled with CytExpert software (Beckman Coulter, Brea, CA, USA). The fluorescence intensity of DCF fluorescein was quantified to assess ROS levels.

**Seahorse assays**

Real-time measurements of oxygen consumption rate (OCR) and extracellular acidification rate (ECAR) were performed using a Seahorse XFe96 Analyzer (Agilent). Macrophages were seeded in a Seahorse XF96 microplate at a density of 1-2 × 10^4 cells of cell culture microplates. Sorted macrophages were derived from WT NEC mice, WT NEC mice, *ACOD1*^-/-^Ctrl mice, and *ACOD1*^-/-^ NEC. 1 hour before analysis, the cells were changed into either Seahorse assay medium (103680100, Agilent) supplemented with 1 mM pyruvate, 2 mM glutamine and 10 mM glucose for mitochondrial stress test or Seahorse assay medium supplemented with 24 mM glutamine for glycolysis stress test. Mitochondrial (103015-100, Agilent) and glycolysis (103020-100, Agilent) stress tests were performed according to the manufacturer’s instructions. For the mitochondrial stress test, the injections were: 1.5 μM oligomycin (oligo), 3 μM FCCP and 0.5 μM of both rotenone and antimycin A (R+A). OCR reads were taken from mitochondrial stress tests and ECAR reads were taken from glycolysis stress tests. OCR and ECAR values were normalized to total protein content. Key parameters were calculated as follows: Basal OCR = Average of three measurements before oligo injection; Maximal OCR = Average of FCCP-induced maximum rate; ATP-linked OCR = (Basal OCR) - (Oligo-inhibited OCR); Proton leak = (Oligo-inhibited OCR) - (R+A-inhibited OCR); Spare Respiratory Capacity = Maximal OCR - Basal OCR; Basal ECAR (mpH/min) = Average of three measurements prior to glucose injection; Glucose-induced ECAR = Maximum rate after glucose injection; Glycolytic Reserve = (Glycolytic Capacity) - (Glucose-induced ECAR); Glycolytic Capacity = Maximum rate after oligo injection; Non-glycolytic Acidification = Average rate after 2-DG injection; Statistical significance was determined by unpaired t-test.

**Tissue collection and injury evaluation**

We initially conducted a precise dissection of the mouse abdomen, and then delicately excised the small intestinal tissue, liver and kidney. Following that, we conducted a thorough initial evaluation of the small intestine and promptly excised the terminal 5 cm segment of the ileum. Subsequently, the terminal 0.5 cm segment of each sample was stabilized in 4 % paraformaldehyde and underwent standard hematoxylin and eosin (H&E) staining. The presence of NEC was confirmed using published tissue injury criteria for necrotic lesions and NEC severity was graded on a 5-point scale according to the published data on NEC mouse models with grade 0: no injury, grade 1: mild separation of lamina propria, grade 2: moderate separation of the submucosa, grade 3: severe separation and/or edema in the submucosa and grade 4: transmural necrosis.

**Immunofluorescence**

The prepared paraffin sections were subjected to a series of critical processing steps to preserve their structural integrity. Initially, deparaffinization was performed, followed by a graded ethanol dehydration series and immersion in ultrapure water. Antigen retrieval was then conducted using a tris-EDTA antigen repair solution under high- temperature conditions. After cooling to room temperature, the sections were extensively rinsed with PBS. Non-specific binding sites were blocked by incubating the sections with 3%–5% BSA for 60 minutes at room temperature. Subsequently, the sections were incubated overnight at 4°C with the appropriate primary antibody. After three washes with PBST, the sections were incubated with the relevant secondary antibodies for 1 hour at room temperature. Nuclear counterstaining was performed using DAPI for 10 minutes at room temperature. The sections were then imaged using a laser scanning confocal fluorescence microscope for further analysis.

**Flow cytometry analysis of apoptosis and JC-1**

After the cells were treated with CCCP, LPS or 4OI, JC-1 staining(Selleck Cat# S9784) was performed according to the kit instructions. CytExpert program software was used to examine apoptosis and JC-1 data.

**Mitochondrial Superoxide Detection and Confocal Imaging**

THP-1 cells were cultured in 35-mm confocal culture dish (Biosharp, Cat# BS-20-GJM), 1x10^5^ cells per dish, and the differentiation was induced by PMA and LPS as described above. At the same time, the cells were treated with 4OI (400ng/ml) and cultured at 37°C in 5% CO_2_. The MitoSOX Red solution was diluted to a working concentration (10μM) and added to dish, incubated at 37°C in the dark for 20min, and images were acquired using a laser scanning confocal microscope (Zeiss LSM900) at an excitation/emission wavelength of 568 nm. The CCCP intervention group was the positive control group. Fluorescence intensity was quantified using ImageJ software.

**Intestinal mucosa permeability assay**

To measure gut mucosal permeability, we administered fluorescein isothiocyanate (FITC)-labeled dextran (FD70; Cat #60842-46-8). Prior to (FITC)-labeled dextran (FD70; Cat #60842-46-8) administration via oral gavage, all mice (both NEC and control groups) were separated from dams and fasted for 4 hours to standardize intestinal contents. During fasting, pups were housed in a temperature-controlled incubator with humidified oxygen. Briefly, all surviving mouse pups at the end of the experiments were gavaged with 750 mg/kg FD70 suspended in sterile PBS (10 mg/mL; Cat #18912014, Thermo Fisher Scientific). After 4 h, pups were sacrificed and plasma levels of FD70 were measured by fluorometry. The dextran concentrations in the plasma of each pup were calculated based on a standard curve

**Enzyme-linked immunosorbent assay**

Plasma samples were collected using EDTA anticoagulant tubes (BKMAMLAB, Cat.No.130201070, China) and analyzed for ITA using an ELISA kit (Jiangsu Meimian Industrial Co., Ltd, Cat. No. MM-927922O2, China). The levels of IL-6, TNF-α and IL-1β were measured using ELISA kits (Ruixin Biotech, Cat. No. RX203049M; Cat. No. RX202412M; Cat. No. RX203063M, Chintable and arrange reagents, samples, and standards. Remove required strips and reseal remaining strips for storage. Add 50 μL of standards, diluent, or samples to appropriate wells, leaving blank wells empty. Add 100 μL of HRP-conjugated detection antibody to all but blank wells. Cover and incubate at 37°C for 60 minutes, protected from light. Discard the liquid, blot dry, wash wells five times, and blot dry again. Mix Substrate A and B (1:1) and add 100 μL to each well. Incubate at 37°C for 15 minutes, protected from light. Add 50 μL of stop solution to each well and measure absorbance using a microplate reader.

**Real-time quantitative reverse transcription polymerase chain reaction**

The total RNA was isolated from ileal tissue using the RNA Quick Extraction Kit (Goonlebio, Cat No.400-100, China), with successful removal of gDNA. The reverse transcription reaction was then performed, followed by the inactivation of reverse transcriptase using the PrimeScript™ RT reagent Kit with gDNA Eraser (Takara, Cat No. RR047A, Japan). The PCR reaction mixture was prepared with the SYBR Premix Ex Taq kit (Takara, Cat No. RR820A, Japan), which included SYBR Premix Ex Taq, primers, DNA template, and ddH_2_O. This mixture was placed in a PCR machine, where pre-denaturation, PCR cycling, and melting curve analysis were conducted according to set protocols. The expression differences between the experimental and control groups were analyzed. As described in previous studies [PMID: 17406449], we utilized the well-established 2^-ΔΔCt^ relative quantification method (Livak & Schmittgen, 2001). The process involves: 1) calculating the mean Ct value from three replicates; 2) normalizing expression levels using the reference gene β-actin (ΔCt = Ct [target gene] - Ct [reference gene]); 3) determining ΔΔCt by comparing with the control group as a baseline; and 4) calculating the final relative expression level using the 2^-ΔΔCt^ formula. The amplification efficiencies of all primers were confirmed through standard curves, ensuring an efficiency range of 90-110%. Statistical analysis was conducted using a two-tailed Student's t-test, with a significance threshold set at p = 0.05. All calculations were performed using GraphPad Prism 9.0. Additionally, the detailed sequences of the reference primers have been added to Appendix Table 2 for clarity.

**Supplementary Figure legend:**

**Supplementary figure 1:**

1. The gating strategy for the Flow cytometry analysis.

(B) Flow cytometry analyze of the percentage of T cells, CD4^+^ T cells, CD8^+^ T cells, B cells, NK cells, neutrophils, macrophages and MDSCs in the spleens of *WT* and *ACOD1^-/-^* mice in NEC group (n=7) and control group (n=10). Data represent mean ± SD, Wilcoxon signed-rank test, Two-tailed. *p* value as shown in Figure.

(C) UMAP plot and heatmap of dimensionality reduction analysis of flow cytometry data for immune cells isolated from intestinal tissues.

(E) Analysis of the Correlation Between NEC Clinical Disease Staging (BELL) and isocitrate, citrate, and cis-aconitate Content. Pearson’s correlation, n=11. r and *p* value as shown in Figure.

**
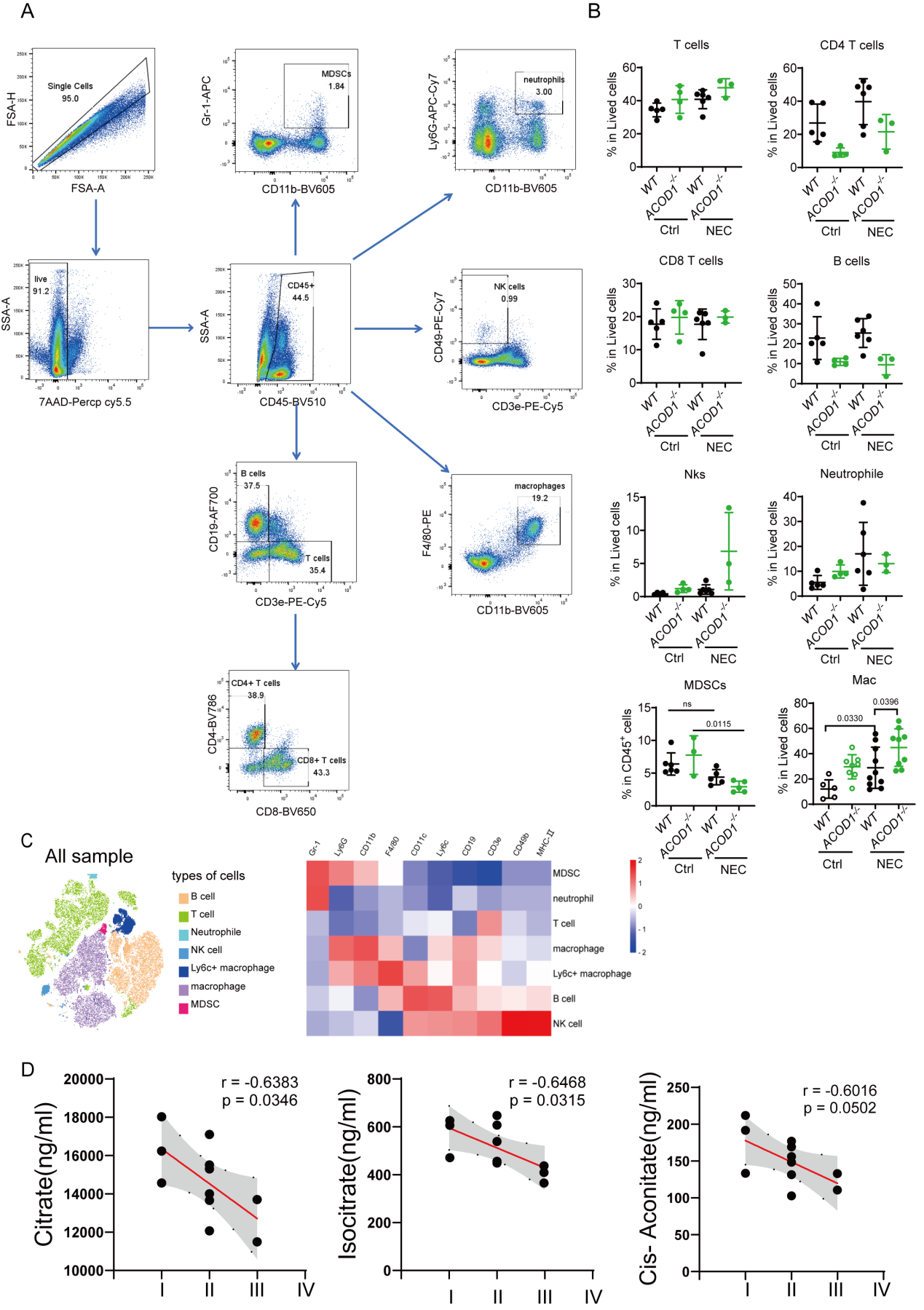
**

**Supplementary figure 2:**

(A) UMAP plot showing 10 cells populations revealed by scRNA-seq.

(B) Cell proportion of 10 cells populations in *WT* mice and *ACOD1^-/-^* mice in NEC group.

(C) The expression of representative signature genes from each cell population was overlaid on the UMAP plot. Color intensity indicates normalized expression levels as shown for each gene.

(D) Scatter plot of *CD68*-positive and CD206-positive cells in the spleen macrophage population of *WT* mice and *ACOD1^-/-^* mice, Data represent mean ± SD, Wilcoxon signed-rank test, Two-tailed, n=5 per group. *p* value as shown in Figure.

(E) Quantitative analysis of *CD68* fluorescence intensity in *WT* mice and *ACOD1^-/-^* mice, Data represent mean ± SD, Wilcoxon signed-rank test, Two-tailed, n=3 per group. *p* value as shown in Figure.

(F) Quantitative analysis of CD86, CD64, CD206 and Arg1 in THP-1 cells induced by 4OI and LPS or IL4 groups. Data represent mean ± SD, Wilcoxon signed-rank test, Two-tailed, n=3 per group. *p* value as shown in Figure.

**
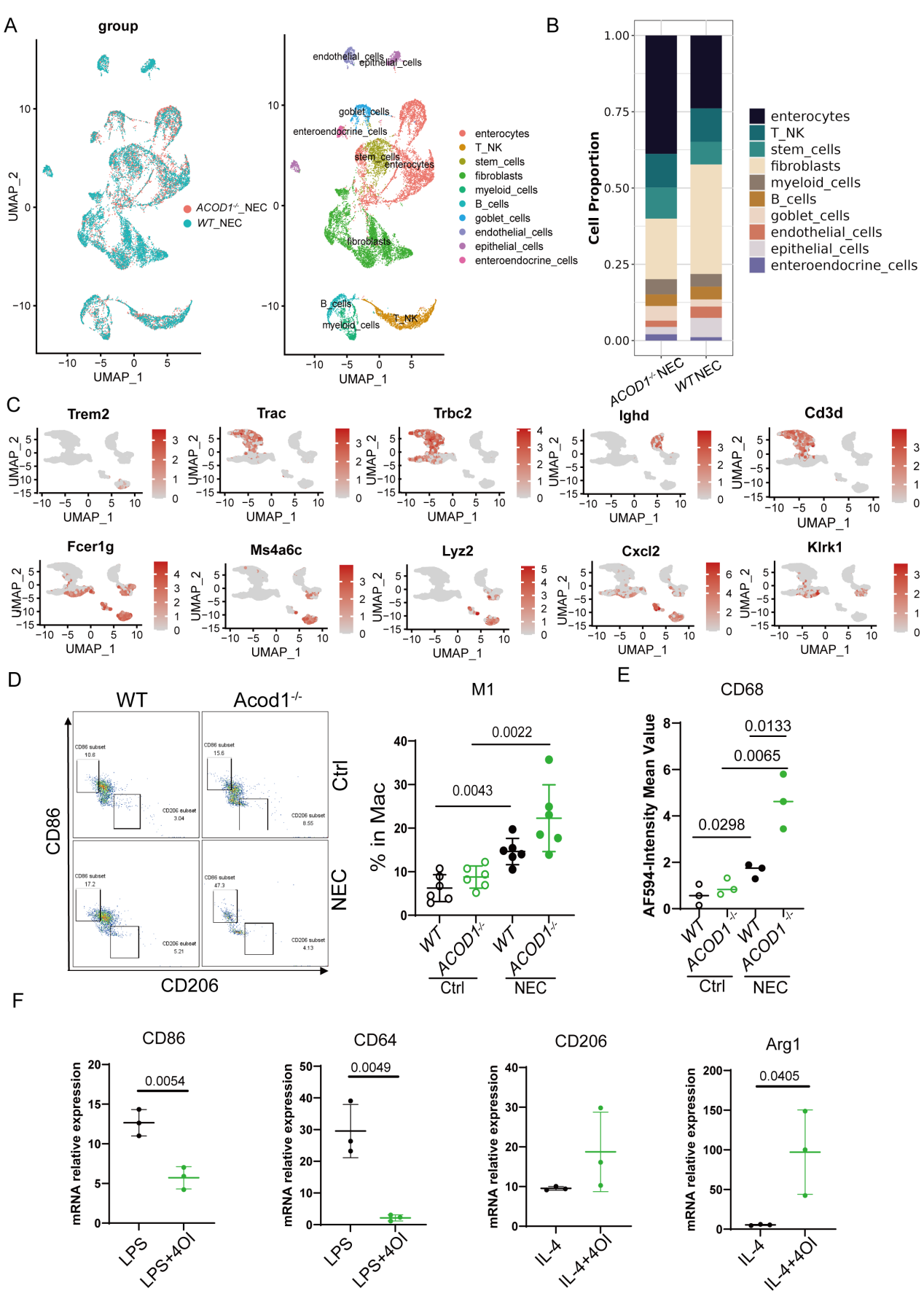
**

**Supplementary figure 3:**

(A) Cnetplot graph constructed from the differential genes and GO enrichment pathways from the Smart-RNA-seq analysis, the size of the dots indicates the percentage of cells expressing the gene of interest, while the intensity of the color indicates expression levels.

(B) The bubble chart illustrating the top 10 pathways at the biological process (BP), cellular component (CC), and molecular function (MF) levels from the GO enrichment analysis of differentially expressed genes.

(C) Cnetplot graph constructed from the differential genes and KEGG enrichment pathways from the Smart-RNA-seq analysis, the size of the dots and intensity of colors as in A.

(D) The bubble chart illustrating the top 18 pathways from the KEGG enrichment analysis of differentially expressed genes.

(E) Top 10 enriched pathways in GO pathway enrichment analysis.

**
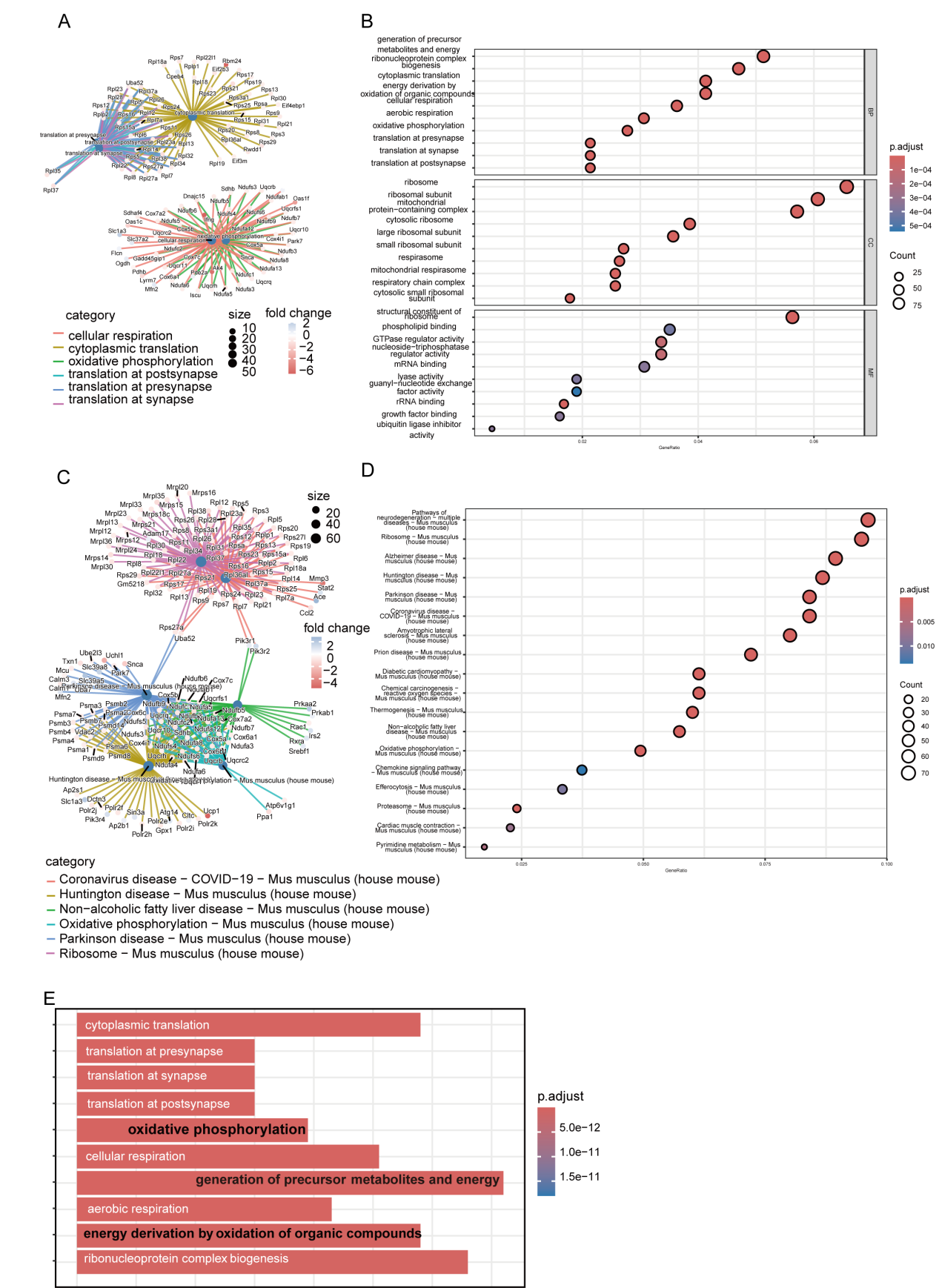
**

**Supplementary figure 4:**

1. Representative images of H&E-stained intestinal epithelial tissue of mice with indicated treatment.

(B) Seversity score and survival curve (C) of NEC mice. Vehicle, vehicle control; ITA, itaconate (n=11 per group).Data represent mean ± SD, Wilcoxon signed-rank test, Two-tailed, *p* value as shown in Figure.

(D) Quantification of expression levels of *IL-6* and *CD68* mRNA (E). The experimental groups were organized as in A. Data represent mean ± SD, Wilcoxon signed-rank test, Two-tailed, *p* value as shown in Figure.

(F) Flow cytometer of ROS and quantitative statistical analysis of MFI in intestinal epithelial tissue of mice with indicated treatment.

(G) Quantitative statistical analysis of CD86, CD64,CD206 and Arg1. Data represent mean ± SD, Wilcoxon signed-rank test, Two-tailed, *p* value as shown in Figure.

(H) MitoSOX Red staining and quantitative statistical analysis of MFI in THP-1, Ctrl n=5, CCCP n=4, LPS n=5, LPS-4OI n=5. Data represent mean ± SD, Wilcoxon signed-rank test, Two-tailed, *p* value as shown in Figure.

**
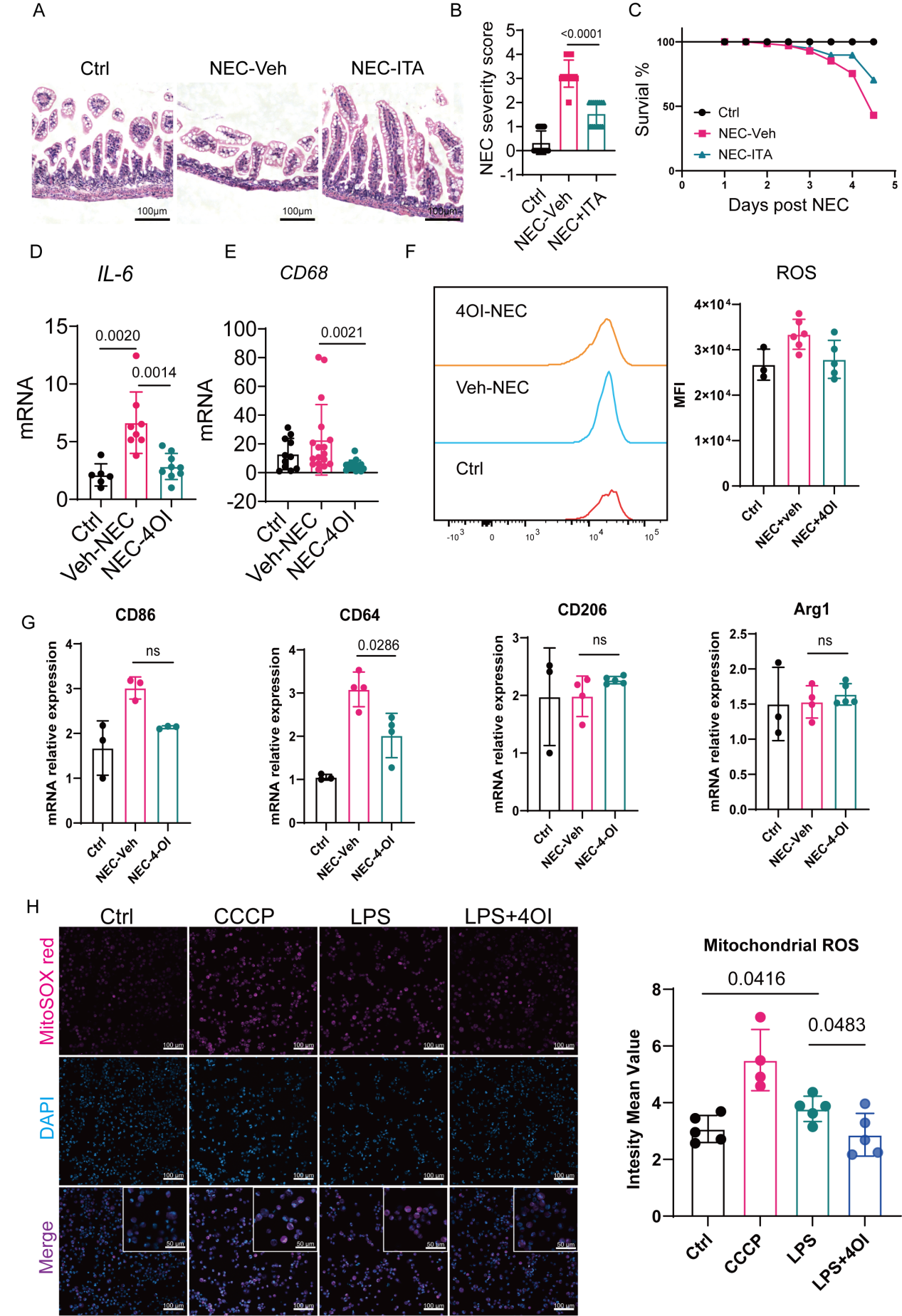
**

**Supplementary figure 5:**

Representative images of H&E-stained kidney (R right, L left) and liver tissue of mice with 4OI treatment. (40mg/kg i.p.)


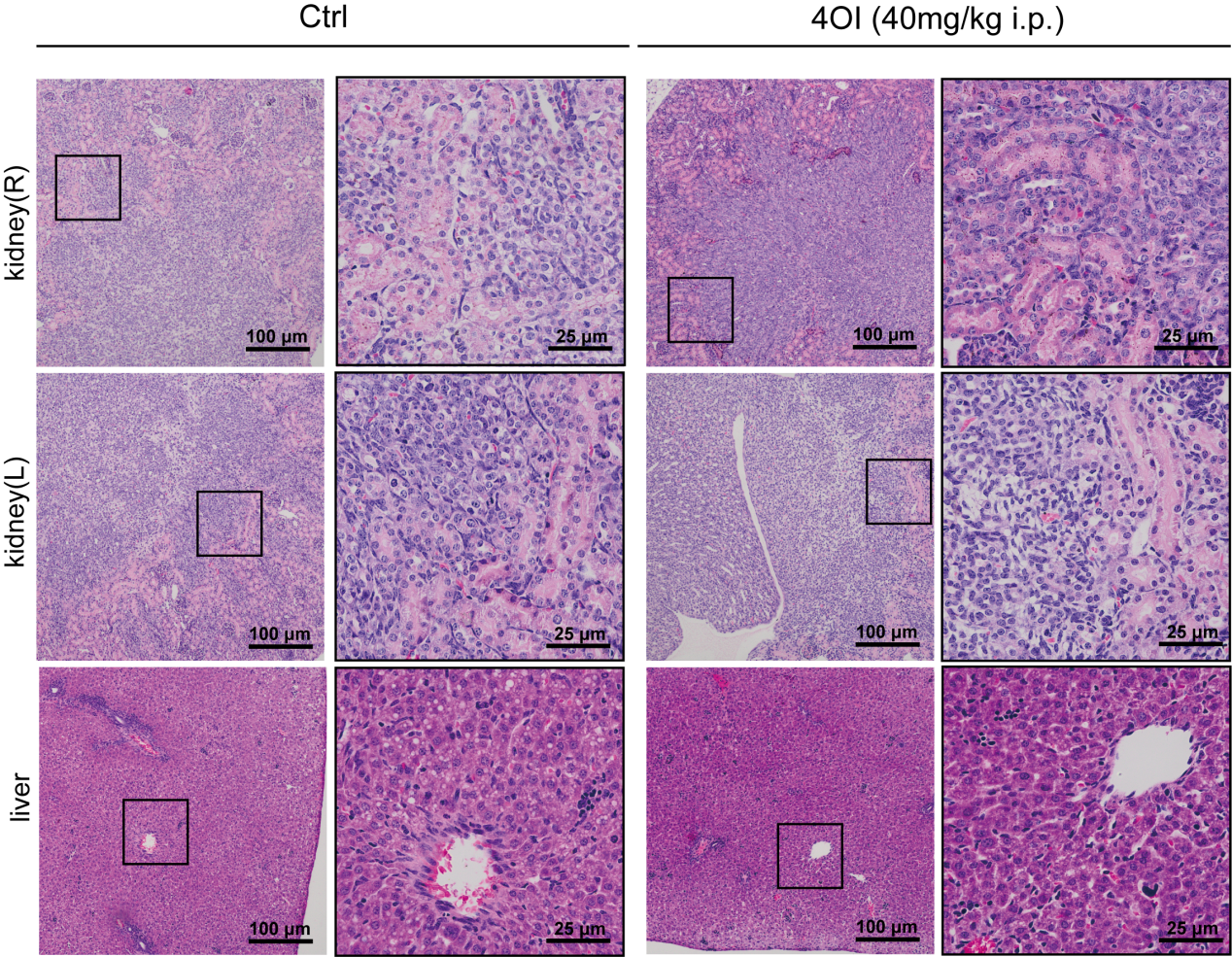


**Key resources table**

| REAGENT or RESOURCE | SOURCE | IDENTIFIER |
| --- | --- | --- |
| Antibodies | | |
| CD3e anti-mouse PE-cy5 | eBioscience | Clone 145-2C11; Cat#15-0031-82 |
| CD4 anti-mouse BV786 | eBioscience | Clone RM4-5; Cat#417-0042-80 |
| CD8A anti-mouse BV650 | eBioscience | Clone 53-6.7; Cat#416-0081-82 |
| CD11b anti-mouse BV605 | eBioscience | Clone M1/70; Cat#406-0112-82 |
| CD11c anti-mouse AF594 | Biolegend | Clone N418; Cat#117346 |
| CD19 anti-mouse AF700 | eBioscience | Clone ID3; Cat#56-0193-82 |
| CD45 anti-mouse BV510 | Biolegend | Clone 30-F11; Cat#103138 |
| CD49b anti-mouse Pe-cy7 | eBioscience | Clone DX5; Cat#25-5971-82 |
| Gr-1 anti-mouse APC | Biolegend | Clone RB6-8C5; Cat#108470 |
| MHC-II anti-mouse BV421 | eBioscience | Clone M5/114.15.2; Cat#404-5321-82 |
| F4/80 anti-mouse PE | Biolegend | Clone BM8; Cat#111604 |
| Ly-6C anti-mouse FITC | Biolegend | Clone HK1.4; Cat#128006 |
| Ly-6G anti-mouse AF780 | eBioscience | Clone 1A8-Ly6g; Cat#56-9668-82 |
| CD86 anti-mouse EF488 | Elabscience | Clone GL-1; Cat# E-AB-F0994L |
| CD206 anti-mouse PE/Cy7 | Elabscience | Clone C068C2; Cat# E-AB-F1135H |
| 7AAD | TONBO | Clone Cat#13-699 |
| Anti-Mouse CD16/CD32 | TONBO | Clone 2.4G2; Cat# 70-0161 |
| Mito-Tracker Green | Beyotime | Cat# C1048 |
| IRG1 Rabbit pAb | Bioss antibodies | Cat# bs-16705R |
| CD68 Monoclonal antibody | Proteintech | Clone 3A9A7; Cat# 66231-2-Ig |
| F(ab')2-Goat anti-Rabbit IgG (H+L) Cross-Adsorbed Secondary Antibody, Alexa Fluor™ Plus 488 | Thermo Fisher | RRID: AB_2896345; Cat# A48282 |
| Donkey anti-Mouse IgG (H+L) Highly Cross-Adsorbed Secondary Antibody, Alexa Fluor™ 594 | Thermo Fisher | RRID: AB_2535789; Cat# A-21203 |
| DAPI | Sigma-Aldrich | Cat# 10236276001 |
| Chemicals, peptides, and recombinant proteins | | |
| 4-octyl itaconate | Selleckchem | Cat# S5929 |
| itaconate | Selleckchem | Cat# SS3095 |
| Dimethyl Sulfoxide | MP Biomedicals | Cat# 196055 |
| Polyethylene glycol 300 | MCE | Cat# 25322-68-3 |
| Tween 80 | MCE | Cat# 9005-65-6 |
| Tween 20 | Biofroxx | Cat# 9005-64-5 |
| PBS buffer (powder) | Life-iLab | Cat# AC08L034 |
| Tris-EDTA antigen repair solution (50X, pH9.0) | Beyotime | Cat# P0084-100ml |
| 0.5M EDTA, pH8.0 | Beyotime | Cat# ST066 |
| Lipopolysaccharides from Escherichia coli O111:B4 | Sigma-Aldrich | Cat# L2630 |
| FD70 | Sigma-Aldrich | Cat #60842-46-8 |
| Glycerol anhydrous | Biofroxx | Cat# 1280ML500 |
| Red Blood Cell Lysis Buffer | Servicebio | Cat# G2015-500ML |
| 2xRealStar Fast SYBR qPCR Mix (Low ROX) | Genstar | Cat# A304-10 |
| BioDewax and Clear Solution | Servicebio | Cat# G1128-500ML |
| Ethanol absolute | Sangon Biotech | Cat# 64-17-5 |
| Environmentally friendly dewaxing clear liquid | Servicebio | Cat# G1128-500ML |
| 3% H_2_O_2_ | Lircon | N/A |
| Hematoxylin Staining Solution | Beyotime | Cat# C0107-100ml |
| DNase/RNase-Free Water | Solarbio | Cat# R1600 |
| PBS, pH7.4 (DNase, RNase & Protease free， Sterile) | Beyotime | Cat# ST478-500ml |
| Sodium Chloride Physiological Solution | Sichuan Kelun Pharmaceutical Co. | N/A |
| Fetal Bovine Serum | Zhejiang Tianhang Biotechnology Co. | Cat# 11011-8611 |
| DMEM/High Glucose (with L-glutamine) | Life-iLab | Cat# AC01L043 |
| HEPES | Sigma-Aldrich | Cat# H3375 |
| Deoxyribonuclease Ⅱ | Biotopped | Cat# D6072C |
| CollagenaseⅣ | Biotopped | Cat# CG6160C |
| Percoll | Cytiva | Cat# 17089101 |
| 10*PBS | Biosharp | Cat# BL551A |
| Corn oil | Beyotime | Cat# ST1177-50ml |
| Isoflurane anesthetic | RWD Life Science | Cat# R510-22-10 |
| JC-1 | Selleck | Cat# S9784 |
| MitoSOX Red | Selleck | Cat# E1376 |
| PMA | Selleck | Cat# S7791 |
| Atovaquone | Selleckchem | Cat# S3079 |
| 4% Paraformaldehyde Fixative | Biosharp | Cat# BL539A |
| Oligonucleotides |  |  |
| See Supplementary table 2 for qPCR Primer Sequences | N/A | N/A |
| Experimental models | | |
| Mouse：C57BL/6 | Zhuhai BesTest Biotechnology Co., Ltd | N/A |
| Mouse：*ACOD1*^-/-^ | Yun Zhao | The Department of General Surgery, BenQ Medical Center |
| Mouse：*ACOD1^fl/fl^* | Cyagen Biosciences | Cat# S-CKO-03141 |
| Mouse：*Lysm^Cre^* | Jackson Laboratory | Cat# 004781 |
| Critical commercial assays | | |
| RNA Quick Extraction Kit | Goonlebio | Cat# 400-100 |
| PrimeScript™ RT reagent Kit with gDNA Eraser | Takara | Cat # RR047A |
| SYBR Premix Ex Taq kit | Takara | Cat # RR820A |
| DAB color development kit | ZSGB Bio | Cat # ZLI-9017 |
| Fluorometric Intracellular Ros Kit | Aladdin | Cat# F486463-1kit |
| Mouse Interleukin 6 (IL-6) ELISA Kit | Ruixin Biotech | Cat# RX203049M |
| Mouse Tumor Necrosis Factor Alpha (TNF-α) ELISA Kit | Ruixin Biotech | Cat# RX202412M |
| Mouse Interleukin 1β (IL-1β) ELISA Kit | Ruixin Biotech | Cat# RX203063M |
| Software and algorithms | | |
| BD LSRFortessa™ X-20 Cell Analyzer | BD | https://www.bdbiosciences.com/en-us/products/instruments/flow-cytometers/research-cell-analyzers/bd-lsrfortessa-x-20; RRID:SCR_025285 |
| FlowJo software (v10.8.1) | BD | <https://www.flowjo.com/solutions/flowo>; RRID:SCR_008520 |
| R (v4.1.3) | R Core Team | [http://www.r-project.org/](http://www.r-project.org/ ); RRID:SCR_001905 |
| GraphPad Prism (v9.5.0) | GraphPad | <http://www.graphpad.com/> ; RRID:SCR_002798 |
| Cell Ranger (v5.1.0) | 10x Genomics | <https://www.10xgenomics.com/support/software/cell-ranger/latest>; RRID：SCRIPT_017344 |
| Seurat (v4.3.0) | N/A | https://seurat.r-forge.r-project.org/; RRID：RRID:SCR_007322 |
| Harmony (v1.0.3) | N/A | https://github.com/immunogenomics/harmony; RRID:SCR_022206 |
| ggplot2 (v3.4.3) | N/A | <https://cran.r-project.org/web/packages/ggplot2/index.html>; RRID:SCR_014601 |
| Complexheatmap (v2.14.0) | Bioconductor | https://bioconductor.org/packages/release/bioc/html/ComplexHeatmap.html; RRID:SCR_017270 |
| QuantStudio^TM^ Design & Analysis SE Software | Thermo Fisher | [https://www.thermofisher.com/order/catalog/product/A28567#/A28567](https://www.thermofisher.com/order/catalog/product/A28567#/A28567) ; RRID:SCR_018712 |
| ZEN 2 (blue edition) | ZEISS | <https://www.zeiss.com/microscopy/en/products/software/zeiss-zen.html>; RRID:SCR_013672 |
| TissueFAXS Viewer(v 7.1.142) | TissueGnostics GmbH | https://tissuegnostics.com/products/scanning-and-viewing-software/tissuefaxs-viewer |
| Chromeleon Chromatography Data System (CDS) | Thermo Fisher | <https://www.thermofisher.com/order/catalog/product/CHROMELEON7>; RRID:SCR_016874 |
| Image J (1.52v) | National Institutes of Health | https://imagej.net/; RRID:SCR_003070 |
| Adobe Photoshop CC (64 Bit) | Adobe | <https://www.adobe.com/products/photoshop.html>; RRID:SCR_014199 |
| Other | | |
| Similac Advance infant formula | Abbott Nutrition | N/A |
| Esbilac puppy milk replacer | PetAg | N/A |
| orthoptic microscope | Olympus | Cat# BX43 |
| Zeiss Axio Imager 2 | ZEISS | https://www.zeiss.com/microscopy/en/products/light-microscopes/widefield-microscopes/axio-imager-2-for-life-science-research.html; RRID:SCR_018876 |
| Bench Type Intelligent Precision Shaker | Shanghai Boxun Medical Biological lnstrument Corp | Cat# BSD-TX345 |
| NanoPhotometer® N60 | IMPLEN | https://www.implen.de/product-page/implen-nanophotometer-n60-microvolume-spectrophotometer/ |
| BD LSRFortessa™ Cell Analyzer | BD | <https://www.bdbiosciences.com/en-us/instruments/research-instruments/research-cell-analyzers/lsrfortessa>; RRID:SCR_018655 |
| BD Biosciences FACSAria III Cell Sorter | BD | <https://www.bdbiosciences.com/en-us/products/instruments/flow-cytometers/research-cell-sorters/bd-facsaria-iii>; RRID:SCR_016695 |
| ZEISS LSM 900 with Airyscan 2 | ZEISS | https://www.zeiss.com/microscopy/us/products/light-microscopes/confocal-microscopes/lsm-900-with-airyscan-2.html; RRID:SCR_022263 |
